# Research progress of DNA methylation on the regulation of substance use disorders and the mechanisms

**DOI:** 10.3389/fncel.2025.1566001

**Published:** 2025-03-31

**Authors:** Ya Liu, Xiao-Qian Wang, Peng Zhang, Abbas Haghparast, Wen-Bin He, Jian-Jun Zhang

**Affiliations:** ^1^Shanxi Key Laboratory of Chinese Medicine Encephalopathy, National International Joint Research Center for Molecular Chinese Medicine, Shanxi University of Chinese Medicine, Jinzhong, China; ^2^Neuroscience Research Center, Institute of Neuroscience and Cognition, Shahid Beheshti University of Medical Sciences, Tehran, Iran

**Keywords:** substance use disorder, DNA methylation, cell-specific, cocaine, opioids, methamphetamine

## Abstract

Drug abuse can damage the central nervous system and lead to substance use disorder (SUD). SUD is influenced by both genetic and environmental factors. Genes determine an individual’s susceptibility to drug, while the dysregulation of epigenome drives the abnormal transcription processes, promoting the development of SUD. One of the most widely studied epigenetic mechanisms is DNA methylation, which can be inherited stably. In ontogeny, DNA methylation pattern is dynamic. DNA dysmethylation is prevalent in drug-related psychiatric disorders, resulting in local hypermethylation and transcriptional silencing of related genes. In this review, we summarize the role and regulatory mechanisms of DNA methylation in cocaine, opioids, and methamphetamine in terms of drug exposure, addiction memory, withdrawal relapse, intergenerational inheritance, and focus on cell-specific aspects of the studies with a view to suggesting possible therapeutic regimens for targeting methylation in both human and animal research.

## 1 Introduction

Substance use disorder (SUD) is a chronic relapsing neuroadaptive disorder characterized by compulsive and uncontrollable pursuit of substances, which is often accompanied negative emotions (e.g., irritability, anxiety) during the withdrawal period (First, 2013). SUD is a global concern that carry significant public health and societal implications (Santos-Toscano et al., 2023). According to the World Drug Report 2024, opioids, methamphetamines and cocaine are most widely used abused drugs. Due to their association with significant morbidity and mortality, we focused on SUDs caused by cocaine, opioid, and methamphetamine abuse.

SUD is accompanied by numerous molecular and biochemical changes (Melo et al., 2020) and is associated with both genetic and environmental factors (Heath et al., 1997; Morozova et al., 2014). Genetic factors influence susceptibility to substances, while epigenetic mechanisms regulate chromatin structure by integrating various environmental stimuli, leading to enduring changes in gene expression and ultimately influencing the development of SUDs (Reilly et al., 2017; Farris and Mayfield, 2021). In addition, a central feature of drug addiction is the high rate of relapse among addicts even after extended periods of abstinence (Berke and Hyman, 2000). This phenomenon is primarily due to the persistence of addiction-related memories. Drug associated cues (conditioned stimulus, CS) are repeatedly matched with the euphoria induced by addictive drugs (unconditioned stimulus, US) to form addiction-related memories. When addicts are exposed to these cues, it triggers the retrieval of addiction-related memories, leading to cravings and potential relapse (Fuchs et al., 2005). The persistence of addiction memories relies on stable alterations in synaptic plasticity in the reward-memory circuits (Kauer and Malenka, 2007; Robbins et al., 2008), which necessitates long-term regulation of gene expression. Emerging research indicates that epigenetic modifications can affect gene transcription and expression in cell cycle, signal transduction and neural plasticity, thereby altering the physiological and pathological functions of brain (Boschen et al., 2018; Mahna et al., 2018).

Epigenetics is the causal mechanisms between genes and gene products resulting in phenotypic outcomes (Waddington, 1942), and epigenetic modifications are defined as certain heritable changes occurring in gene function without affecting the nucleotide sequence (Goldberg et al., 2007; Berger et al., 2009). Epigenetic modifications are broadly classified into four major categories: DNA methylation, chromatin remodeling, non-coding RNA, and histone modifications (Weinhold, 2006). One of the most well-studied forms of epigenetic modification is the methylation of cytosine at CpG sites. This process plays a crucial role in mediating specific changes in gene promoters over time and space, and these changes are potentially reversible (Stevenson and Prendergast, 2013; Kim and Costello, 2017). DNA methylation can be influenced by various factors, including environmental stress, chemical exposure, diseases, and cancer (Vidaki et al., 2013). Abnormal DNA methylation patterns have been observed in the promoter regions of several genes in drug addicts and animal models. Exposure to drugs can lead to either hypermethylation or hypomethylation in these gene promoter regions, which in turn affects gene expression. Studying DNA methylation offers a comprehensive understanding of epigenetic gene regulation (Moore et al., 2013).

DNA methylation has long been thought to permanently silence genes in the brains (Day and Sweatt, 2011). However, some studies have revealed the existence of active demethylation catalyzed by the TET (ten-eleven translocation) family of enzymes in mammals. This has prompted researchers to rethink how DNA methylation influences neuronal long-term plasticity, both methylation and demethylation, may collaborate to regulate the expression of key genes (Tahiliani et al., 2009; Ito et al., 2011). It should also be noted that these findings are not in contradiction with the heritability of DNA methylation. After several decades of studies, our understanding of DNA methylation has been expended. DNA methylation formations are relatively conserved in mammals, but the DNA methylation states vary in the various tissues and according to the growth stages and can be directly modulated by environmental stimuli. It is clear that DNA methylation act as an inherited epigenetic marker (Wang et al., 2017). The constitutive DNA methylation can be stably inherited, while the DNA methylation induced by stimuli (including drug) is dynamic. Interestingly, some of these induced DNA methylations may transfer to inherited, via change the DNA methylation in gamete (Nestler, 2014). In particular, although studies found that DNA methylation in male and female germlines is largely eliminated from the genome during gametogenesis and after fertilization, it is ascertained that the erasure of DNA methylation during two waves of genome-wide reprogramming is not complete (Guibert et al., 2012; Seisenberger et al., 2012; Hackett et al., 2013). The methylation status of cytosine can escape erasure following fertilization with the help of Dnmt1, leading to largely maintained at differentially methylated regions of imprinted genes (Wang et al., 2017).

Particularly, in SUD, like in other psychiatric disorders, DNA methylation patterns seem to display duality: reversibility and hereditability, which offers valuable insights into how substance abuse affects individuals and their offspring. Dynamic methylation modification in somatic cells, like the responses in neurons to drug exposure, contrasts with stable modifications found in germ cells, such as imprinted genes. This dual mechanism allows organisms to quickly adjust gene expression in response to abused drugs or related motivation while preserving the integrity of genetic information. Short-term drug exposures may lead to temporary DNA methylation, which can often be reversed by demethylation, restoring the original methylation patterns. However, prolonged drug use may exceed the limits of this homeostasis, resulting in irreversible methylation reprogramming in germ cells. This reprogramming can impact the individual health and may also be passed on to future generations. Interestingly, certain methylation patterns remain highly conserved throughout evolution. For example, the methylation patterns involved in transposon silencing are crucial for maintaining genomic stability. These inherited methylation patterns contribute to genomic stability, while dynamic modifications of DNA methylation enable adaptation to short-term environmental changes. This interplay between stability and dynamism reflects an organism’s delicate balance between genetic and environmental influences, highlighting the ongoing relationship between DNA methylation and demethylation.

This review highlights DNA methylation alterations observed in gene promoter regions and other active loci from five perspectives including drug exposure, addiction related memory, withdrawal and relapse, intergenerational inheritance, and cellular specificity, and the discussion focuses on a concise description of DNA methylation and DNA demethylation alterations, particularly in response to cocaine, methamphetamine (METH), and opioids, as observed in human and animal studies (Table 1).

**TABLE 1:** Multidimensional profiling of the DNA methylation alterations of target genes induced by major psychoactive drugs.

Different perspectives		Cocaine	References	Opiates	References	Methamphetamine	References
Drug exposure	Acute	PP1C↑	Anier et al., 2010	OPRM1↑	Sandoval-Sierra et al., 2020		
	Chronic	PP1C↑	Anier et al., 2010	OPRM1 ↓	Agulló et al., 2024	CHN2↑	Hao et al., 2017; Nestler, 2013
		Cdkl5↑	Carouge et al., 2010	Netrin-1↑	Shu et al., 2021	SHATI/NAT8L↑	Yuka et al., 2020
		CREM↑	Ajonijebu et al., 2018	HTR1B ↑	Li et al., 2022	BDNF↑	Salehzadeh et al., 2020; Iamjan et al., 2021
		FosB↓	Ajonijebu et al., 2018	Bdnf IV↓	Chao et al., 2016	Crh↓, Avp↓	Jayanthi et al., 2018
		Homer2↓	Ploense et al., 2018			GluA1↓, GluA2↓	Jayanthi et al., 2014
Addiction memory	SA	CREB↑	Massart et al., 2015	CaMKK1↓	Chen et al., 2021; Jiang et al., 2021		
	CPP	Cdkl5↑	Bodetto et al., 2013	Arc↓, Dlg1↓, Dlg4↓, Syn1↓,	Fan et al., 2019; Fan et al., 2019		
		Bdnf IV↓	Tian et al., 2016				
Withdrawal and relapse		Golgb1↑	Alaghband et al., 2016	OPRM1↓	Camerota et al., 2022	Kcna1 ↓, Kcna3 ↓, Kcnn1↓	Jayanthi et al., 2020
		OXTR↑	Souza et al., 2023			SNCA↓	Biagioni et al., 2019
Intergenerational inheritance	Father	Cdkn1a↓	Swinford-Jackson et al., 2022	OPRM1↑	Chorbov et al., 2011	HSD11B2↑	Oni-Orisan et al., 2021
	Newborn			OPRM1↑	Wachman et al., 2014		
Cell specificity	Oligodendrocyte	Sox10↓	Nielsen et al., 2012a				
	Microglia	pri-miR-124a-1↑, pri-miR-124-2↑	Guo et al., 2016				
	Astrocyte	mtDNA↓	Doke et al., 2021				
	GABAergic neurons					PVALB↑	Veerasakul et al., 2017
	Leukocyte			OPRM1↑	Ebrahimi et al., 2018; Doehring et al., 2013		

PP1C, protein phosphatase 1 catalytic subunit gamma; Cdkl5, cyclin-dependent kinase-like 5; CREM, cAMP-responsive element modulator; FosB, FBJ murine osteosarcoma viral oncogene homolog B; Homer2, homer protein homolog 2; CREB, cAMP-response element binding protein; Cdkl5, cyclin-dependent kinase-like 5; Bdnf IV, brain-derived neurotrophic factor (BDNF) transcripts containing exon IV (Bdnf IV); pri-miR-124a-1, primary microRNA 124a-1; Golgb1, golgin B1; OXTR, oxytocin receptor; Cdkn1a, cyclin-dependent kinase inhibitor 1A; Sox10, SRY-box transcription factor 10; mtDNA, mitochondrial DNA; pri-miR-124-2, primary microRNA 124-2; OPRM1, μ-opioid receptor gene; Netrin-1, netrin-1; HTR1B, 5-hydroxytryptamine receptor 1B; CaMKK1, calcium/calmodulin-dependent protein kinase kinase 1; Arc, activity-regulated cytoskeleton-associated protein; Dlg1, disks large homolog 1; Dlg4, postsynaptic density protein 95(PSD-95); Syn1, synapsin I; CHN2, chimerin 2; NAT8L, N-acetyltransferase 8-Like; Crh, corticotrophin releasing hormone; Avp, vasopressin; GluA1, glutamate ionotropic receptor AMPA type subunit 1; GluA2, glutamate ionotropic receptor AMPA type subunit 2; Kcna1, potassium voltage-gated channel subfamily A member 1; Kcna3, potassium voltage-gated channel subfamily A member 3; Kcnn1, potassium calcium-activated channel subfamily N member 1; SNCA, alpha-synuclein; HSD11B2, 11β-hydroxysteroid dehydrogenase type 2; PVALB, parvalbumin. ↑ indicates DNA methylation; ↓ indicates DNA demethylation.

## 2 Epigenetic modification of DNA

DNA methylation is a significant epigenetic marker involved in the life processes of eukaryotes (Zhang et al., 2018). In vertebrates, DNA methylation underlies regulatory mechanisms such asembryonic development and cell reprogramming (Planques et al., 2021). Epigenetic modification of DNA refers to changes in DNA sequences caused by external environmental stimuli without altering the organism’s underlying DNA sequence. This includes DNA methylation and DNA demethylation processes. Such modification serves as a signaling system that encodes past experiences and integrates present experiences, and is able to regulate gene transcription and thus neuronal function, which in turn affects behaviors.

DNA methylation is facilitated by DNA methyltransferases (DNMTs), which transfer a methyl group of S-adenosylmethionine (SAM) to the fifth carbon atom of cytosine to form 5-methylcytosine (5mC), participating in long-term silencing of genes (Kulis and Esteller, 2010). More than 80% of CpG sites in the human genome are scattered and highly methylated (Morris and Monteggia, 2014). The 0.5–5 kb DNA fragments with CpG dinucleotide clusters in the 60%–70% GC-rich DNA region are designated as CpG islands, which are located in the first exon and promoter of the gene (Takai and Jones, 2003). CpG islands are usually unmethylated and highly conserved, and about 70% of promoters contain CpG islands. CpG island methylation in promoter region regulates gene transcription through multiple mechanisms (Saxonov et al., 2006). For example, the genes transcription is suppressed by blocking the binding of transcription factors (TFs) to the promoter region, or inhibition of transcription by Methylated CpG site-binding proteins to recruit co-inhibitory complexes (Héberlé and Bardet, 2019).

### 2.1 DNA methylation

DNA methylation is catalyzed and maintained by DNMTs. The three main isoforms are DNMT1, DNMT3a and DNMT3b. DNMT3A and DNMT3B are enzymes that establish initial methylation patterns on unmethylated DNA and play an important role in early embryonic development and normal cellular differentiation (Greenberg and Bourc’his, 2019), while DNMT1 is mainly responsible for maintenance of methylation, which means that after one of the DNA strands is methylated, DNMT1 catalyzes the methylation of the other strand. This allows DNMT1 to recognize the methyl group of one of the DNA strands even if it is removed, and to keep the methylation level stable by methylating this site.

DNMT1 replicates methylation patterns from parent DNA strands to progeny strands during DNA replication (Turek-Plewa and Jagodziński, 2005). DNMT1 knockout in mice leads to DNA methylation loss, cell apoptosis, and embryonic death (Moore et al., 2013). Another DNMT, DNMT3L, is expressed only in adult germ cells and early developing thymus. DNMT3L is a non-catalytic protein that combines DNMT3A and DNMT3B with methyltransferase activity. In mice, DNMT3L participates in establishing genomic imprinting, reverse methylation transfer, and X chromosome agglutination between offspring and parents (Aapola et al., 2000; Hata et al., 2002).

Methylation of CpG sites in the promoter can be specifically recognized by methylated CpG binding domains (MBDs), including MBD1-4 and methylated CpG binding protein 2 (MeCP2)(Sadri-Vakili, 2015). MBD contains transcriptional inhibition domains that bind to various repressor complexes to inhibit transcription (Sarraf and Stancheva, 2004). MeCP2 can recruit DNMT1 to semi-methylated DNA to maintain methylation (Sarraf and Stancheva, 2004). MeCP2 also binds to CpG sites and recruits transcription inhibitor complexes, such as DNMTs and histone deacetylases (HDACs), thus inhibiting gene transcription (Sadri-Vakili, 2015).

### 2.2 DNA demethylation

DNA methylation was once thought to be a stable genetic marker that was heritable and could not undergo demethylation. However, a study has shown that both passive and active demethylation can occur. During cell division, passive demethylation is achieved by inhibiting DNMT1 expression or catalytic activity and diluting/reducing the density of methylated cytosine in the genome (Tahiliani et al., 2009). The majority of DNA is passively demethylated during cleavage. Active DNA demethylation is mainly dependent on the TET family (ten-eleven translocation enzymes, TET1/2/3) and thymine-DNA glycosylase (TDG). The TET enzyme oxidizes 5mC and 5-hydroxymethylcytosine (5hmC) to 5-formylcytosine (5fC) and 5-carboxylcytosine (5caC), while TDG is responsible for the selective identification and removal of 5fC and 5caC, restoring them to cytosine by base excision repair.

Studies have suggested that 5-hmC is not only a mediator of DNA demethylation but also functions as a stabilizing marker in epigenetics, playing a crucial role in regulating gene expression (Szulwach et al., 2011; Song et al., 2013, Li et al., 2015). There are three TET enzymes identified in mammals, TET1, TET2, and TET3. TET and its catalytic product 5-hmC are abundant in the brain, and the levels of 5-hmC are rapidly and reversibly altered in genes related to synaptic plasticity and memory, suggesting that TET play a critical role in neural activity, especially memory processing (Sultan et al., 2012; Kaas et al., 2013; Rudenko et al., 2013). Compared to TET1 and TET2, TET3 has higher expression level in the cortex and hippocampus - key brain regions for memory processing, suggesting dissociation among different subtypes of TET (Alaghband et al., 2016).

DNA demethylation influences specific memory circuits related to addiction by regulating gene expression which contribute to neuroplasticity. Active DNA demethylation is a result of neuronal activity and TET can decrease methylation levels at memory and synaptic plasticity associated genes (Miller and Sweatt, 2007; Miller et al., 2008; Guo et al., 2011; Li et al., 2013). Specifically, TET1 in the dentate gyrus (DG) enhances the expression of brain-derived neurotrophic factor (BDNF) by inducing DNA demethylation at the BDNF promoters, which in turn supports reward memory retrieval (Sagarkar et al., 2022). Cocaine exposure negatively affects the expression of TET1 in the NAc, resulting in a specific accumulation of 5-hmC in enhancer and gene coding regions. These dynamic modifications are linked to alternative splicing and the reprogramming of gene expression, potentially strengthening and prolonging drug reward memory by influencing the activity of genes associated with synaptic plasticity, such as addiction-related transcription factors and signaling molecules. Furthermore, TET3 was dramatically upregulated after retrieval of cocaine-related memory, and knocking down TET3 in the dorsal hippocampus inhibits the activation of pyramidal neurons and disrupts the reconsolidation of cocaine-related memories (Liu et al., 2018). TET3 knockdown also decreases DNA hydroxymethylation, impairs the dendritic spine density, PSD length, and thickness of neurons, reduces the expression of synaptic plasticity-related genes including Homer1, Cdkn1a, Cdh8, Vamp8, Reln, Bdnf, while surprisingly increases immune-related genes Stat1, B2m, H2-Q7, H2-M2, C3, Cd68. Notably, knockdown of TET3 in NAc activates microglia and CD39-P2Y12R signaling pathway (Fan et al., 2022). All these findings suggest that DNA demethylation may modulate addiction related brain regions via regulating synaptic plasticity and immune-related signaling pathway.

## 3 Cocaine and epigenetic modification of DNA

Cocaine, a psychostimulant, blocks the monoamine transporters of the plasma membrane because of which dopamine clearance from the synaptic cleft in the brain gets inhibited (Joffe et al., 2014). Hippocampal pyramidal neurons, prefrontal cortex (PFC), striatum, and NAc have exhibited altered DNA methylation patterns in response to cocaine intake (Novikova et al., 2008; LaPlant et al., 2010).

### 3.1 Drug exposure

Acute cocaine exposure leads to transient changes in the activity or expression levels of DNMTs in PFC and nucleus accumbens (NAc), which may affect the methylation status of specific genes. Acute cocaine administration increases DNA methylation as well as the expression of DNMT3A and DNMT3B in the NAc (Anier et al., 2010). LaPlant et al. (2010) reported that DNMT3A mRNA was up-regulated at 4 h but down-regulated at 24 h after a single cocaine injection. An isoform of DNMT3A, DNMT3A2, was overexpressed in NAc shell but not NAc core in rats, has been reported following acute cocaine administration (Cannella et al., 2018). Cocaine also increased DNA hypermethylation and increased binding of methyl CpG binding protein 2 (MeCP2) at the protein phosphatase-1 catalytic subunit (PP1c) promoter associated with decreased PP1C mRNA levels (Anier et al., 2010). So, Increased DNA methylation following acute cocaine is associated with enhanced binding of MeCP2 to specific gene promoters and corresponding decreases in gene transcription (Anier et al., 2010). In addition, although there is relatively little evidence for direct demethylation under acute exposure, it is hypothesized that cocaine may work by affecting the activity or expression of demethylating enzymes. For example, acute cocaine treatment decreases transcript levels of TET1 and TET2 in the NAc in mouse (Anier et al., 2018).

These changes may pave the way for chronic exposure and the development of addiction. With prolonged cocaine exposure, the brain begins to undergo a series of adaptive changes that involve multiple levels of neurotransmitter systems, neural circuits, and epigenetic mechanisms. Chronic cocaine exposed leads to significant alterations in DNA methylation levels, which are often highly correlated with addiction-related behavioral manifestations. A study suggests that chronic exposure to cocaine in rats and mice causes hypermethylation in the striatum and hypomethylation in the PFC (Tian et al., 2012).

Repeated cocaine administration has been shown to alter the transcription of DNMT3a (but not DNMT3b) in the mouse NAc (LaPlant et al., 2010). In contrast, another study found that repeated cocaine injections increased mRNA levels of DNMT1, DNMT3A, and DNMT3B in the NAc (Anier et al., 2018). Additionally, repeated cocaine resulted in DNA hypermethylation and increased MeCP2 binding to the PP1c promoter, resulting in downregulation of the PP1c gene (Anier et al., 2010), as was seen with Cdkl5 (Carouge et al., 2010). Furthermore, in the NAc, which is highly implicated in the motivational aspects of drug-seeking, repeated cocaine injections leads to increases in dendritic branching and dendritic spine formation (Robinson and Kolb, 1999; Dumitriu et al., 2012).

The increase in DNA methylation levels in the NAc and the decrease in DNA methylation levels in the PFC may be related to an attenuation of the reward effect and an enhancement of compulsive medication behavior. Studies conducted outside the NAc have demonstrated that the expression of Dnmt transcription is affected by cocaine exposure in the extended mesocorticostriatal dopamine pathway. In the hippocampus, Dnmt3a expression increases after a brief withdrawal from chronic cocaine injections, while Dnmt3b levels remain elevated for up to 24 h following a single dose (Anier et al., 2010).

Cocaine-associated methylation changes in specific gene promoters can exhibit variations across distinct models and brain regions. In a chronic cocaine exposure mouse model, DNA methylation at the promoter regions of Fosb and Crem (cAMP response element modulator) was significantly reduced in the prefrontal cortex (PFC) compared to controls, whereas methylation levels at the same promoters in the hippocampus (HPC) were significantly elevated, indicating that cocaine induces brain region-specific methylation reprogramming to regulate gene expression (Ajonijebu et al., 2018). Furthermore, in a transgenerational epigenetic model, parental cocaine exposure led to decreased Crem and Fosb promoter methylation in the PFC of progenitor mice, and similar methylation patterns were replicated in the brains of drug-naive offspring (Ajonijebu et al., 2019). These studies collectively suggest that cocaine-associated alterations of DNA methylation are highly precise and controllable, and further studies need to explore the upstream signal pathway that induces DNA methylation in the brain. These mechanisms are largely unknown.

DNA demethylation may also play an important role during the chronic cocaine exposure. By removing methyl groups, demethylation can restore the expression of originally silenced genes, thus participating in the cocaine addiction. TET1, but not TET2 or TET3, has been demonstrated to be downregulated in the NAc following repeated cocaine administration. Further, differential levels of 5-hydroxymethylcy cytosine were reported at 11,511 regions across autosomes following repeated cocaine exposure (Feng et al., 2015). Studies have investigated DNA hydroxymethylation changes cocaine administration Increased global levels of 5-hmC were noted after repeated cocaine exposure (Anier et al., 2018). Ploense et al. (2018) found that limited access to cocaine decreased DNA hydroxymethylation within the Homer2 promoter in rats. Cocaine-induced behavioral sensitization is associated with increased expression and activity of DNMTs and decreased expression and activity of TET1 and TET3 in mouse NAc (Anier et al., 2018). These changes are correlated with altered 5-mC and 5-hmC levels at the candidate gene promoter regions. These genes might regulate relapse behavior by affecting the consolidation and extraction of addiction-related memories.

Specific intervention treatments may directly affect DNA methylation processes in cocaine exposure. Systemic administration of the methyl donor SAM modulate the level of cocaine-induced DNA methylation in the NAc (Anier et al., 2013). After chronically injecting mice with cocaine, Anier et al. (2013) performed an expression microarray study in the NAc, generating lists of significantly up-regulated and down-regulated genes. Interestingly, the authors found that that repeated SAM treatment reversed the hypomethylation of the gene Gal but also unexpectedly reduced the hypermethylation of the gene Slc17a7. In addition, they observed decreased promoter methylation of the Dnmt3a and Dnmt3b genes, which coincided with increased expression of these genes. Pharmacologically blocking DNMT activity decreased cocaine-induced PP1c hypermethylation and gene expression changes while delaying the development of cocaine-induced behavioral sensitization while the opposite effect was seen at the immediate early gene (Anier et al., 2010). Therefore, cocaine does not appear to cause global changes in DNA methylation non-specifically. Instead, specific genes or networks of genes appear to be co-regulated at the level of chromatin following drug exposure.

### 3.2 Addiction-related memory

It is well documented that environmental cues associated with drug use can enter an association with the rewarding effects of drugs, becoming addiction-related memory. DNA methylation may influence the formation and maintenance of addictive memories by modulating the expression of specific genes within the NAc and PFC. In self-administration (SA) models, animals actively acquire cocaine through SA behavior, resulting in the formation of addiction related memories. Cocaine alter DNA methylation patterns in the brain by affecting DNA methyltransferase activity. These alterations involve the regulation of the expression of addiction-related genes, thereby affecting the formation and maintenance of addiction memories. A study found that mice self-administered cocaine showed significant decreases in DNMT1 and DNMT3a mRNAs measure 24h after the last session (LaPlant et al., 2010). Using rats that self-administered cocaine, Wright et al. (2015) found significant increases of DNMT3A in the NAc but not in the PFC. Of related interest, rats that underwent cocaine SA also exhibited increased MeCP2 expression in the dorsal striatum (Im et al., 2010). Changes in gene expression following cocaine SA also correlate with increased expression of MeCP2 (Host et al., 2011). Using genome-wide sequencing, Baker-Andresen et al. (2015) found that rats that self-administered cocaine exhibited 29 differentially methylated regions in a persistent manner in comparison to saline- or passive cocaine-treated animals, with five regions being demethylated and the rest hypermethylated.

A study measured methylation of more than 40 genes of interest by microarray design and detected differential patterns of DNA methylation after cocaine SA using a candidate gene approach. In this study, the authors detected a correlation between increased DNA methylation at specific loci (e.g., CREB) and reduced expression of those genes in response to drug exposures (Massart et al., 2015). Furthermore, the process of demethylation may be involved in the reversal or abrogation of addiction memories. By removing methyl groups, demethylation can restore or alter the expression patterns of specific genes, thereby attenuating or eliminating addiction memories. In a PFC study, Fonteneau et al. (2017) trained rats to self-administer cocaine, either alone or after intra-ventricular injections of DNMT inhibitors. As was seen in mice, SA resulted in numerous DMRs, most of which fell within gene bodies and intergenic regions. Interestingly, the DNMT inhibited group also showed more hyper- than hypomethylation in response to cocaine SA, which suggests that, at least in the PFC, cocaine-related hypermethylation is more related to a decreased removal of methylation rather than an addition of methyl groups to new locations.

In the conditioned position preference (CPP) model, passive and involuntary intake of cocaine tends to induce the same effect on the brain reward and memory system. For example, Cdkl5 DNA methylation represses the β- subunit of protein phosphatase-1 (PP1Cβ) in response to cocaine exposure in the rat brain by induction of MECP2 expression influencing the learning and memory, whereas the α-subunit of protein phoshatase- 1 (PP1Cα) levels remain unchanged (Bodetto et al., 2013). It has also been suggested that altered levels of DNA methylation in the hippocampus affects the acquisition and recovery of cocaine induced CPP in mice. Han et al. (2010) used 5-aza-2-deoxycytidine (5-aza), to prevent DNMT and examine the effects on the acquisition and retrieval of cocaine induced CPP. They found that inhibiting Dnmt in the hippocampus of C57BL/6 mice prior to training impaired their ability to acquire CPP, whereas the same manipulations in the prelimbic cortex prevented the retrieval of the conditioned memory after a 24-h delay. Conversely, systemic injections of the methyl donor L-methionine before and throughout training, reversed the establishment of cocaine CPP (Tian et al., 2012).

A study of gene-specific methylation changes in the striatum investigated changes at the Bdnf locus (Tian et al., 2016). The authors found an increased expression of a specific isoform of this gene, Bdnf transcripts containing exon IV (Bdnf IV), in the NAc of mice after CPP training. Notably, this change appears to be specific to CPP itself, as there was no difference in its expression in non-conditioned, cocaine treated animals. After bisulfite sequencing, the authors identified hypomethylation at a single CpG site within the promoter region of Bdnf IV that overlapped with the binding site for the C-myb transcription factor. Despite the methylation difference occurring at a single nucleotide, the authors demonstrated increased C-myb binding at the Bdnf IV promoter in cocaine-conditioned animals (Tian et al., 2016). This, along with data suggesting that Bdnf overexpression increases cocaine consumption (Im et al., 2010), provides convincing evidence for a pathway between DNA methylation, neurotrophic signaling, and cocaine-related behaviors in this brain region. In summary, these transcription factors and proteins may affect gene expression activity by binding to DNA methylation or demethylation sites.

### 3.3 Withdrawal and relapse

Repeated drugs use forms drug-related memories and causes long-lasting neural changes in the brain, which contribute to a high risk of relapse even after long-term withdrawal. Altered DNA methylation patterns may persist during withdrawal, leading to cocaine craving and relapse behavior in addicts.

Interestingly, when DNMT3a is knocked down prior to abstinence, the authors observed an increase in cocaine preference during CPP, this suggests that the increase in DNMT3a during abstinence could serve as a neuroprotective mechanism that counters addiction-related changes in the brain. Roles for DNMT3a during drug abstinence and relapse were investigated utilizing an operant cocaine SA paradigm. In this study, the authors did not identify immediate changes in DNMT3a expression following cocaine SA, but when animals were put through forced abstinence, significant increases in DNMT3a expression were observed beginning 2 days after the last cocaine infusion. These findings raise the possibility that increased DNMT3a activity during withdrawal may serve as an epigenetic mechanism important for potentiating relapse vulnerability. Knockdown of DNMT3a in the NAc was found to significantly reduce rates of cue-induced reinstatement (Cannella et al., 2018), suggesting that DNMT3a overexpression may indeed be a key driver of drug-seeking behaviors. Preference for cocaine was attenuated by pharmacological inhibition using a DNMT inhibitor, RG108 (LaPlant et al., 2010). The persistent induction of DNMT3a after a month of cocaine abstinence could be crucial for understanding molecular susceptibility to relapse and merits further investigation for potential therapeutic interventions. Additionally, it is important to note that different subtypes of DNMTs have distinct roles. Specifically, Dnmt3a2, but not Dnmt3a1 in the NAc is required for relapse in cocaine SA rats (Cannella et al., 2018).

Massart et al. (2015) reported that NAc micro-injections of RG108 or SAM, decrease and increase, respectively, cocaine seeking on abstinence day 30, and that these effects last for up to abstinence day 60. Cannella et al. (2018) extended these findings and examined the role of a specific DNMT isoform in NAc, DNMT3A2, in incubation of cocaine craving. First, they reported that viral knockdown of DNMT3A2 by shRNA in NAc shell during abstinence or prior to cocaine SA decreases cue-induced reinstatement on abstinence day 45. In the latter condition, DNMT3A2 knockdown also decreases cue-induced reinstatement on abstinence day 1 but has no effect on cocaine SA. These data indicate that DNMT3A2 in NAc plays a role in cocaine relapse.

A study utilized methyl-binding protein immunoprecipitation and high-throughput sequencing (MBD-seq) to analyze the methylation patterns in the mouse prefrontal cortex (PFC) after either cocaine SA or injection, followed by periods of acute or prolonged abstinence and a relapse test (Baker-Andresen et al., 2015). They found distinct methylation enrichment patterns for each condition, and deferentially methylated regions (DMRs) that were associated with cocaine-seeking after prolonged abstinence. In general, DMRs were enriched in gene bodies and were more methylated in the cocaine group, although there were distinct loci that appeared less methylated. Importantly, only one of their validated DMRs, Golgb1, a Golgi-related transport protein, corresponded with changes in the overall gene expression, and both measures were decreased in cocaine animals (Alaghband et al., 2016).

In the PFC, one study found increased Dnmt3a mRNA expression and decreased Dnmt3b mRNA and protein, shortly after SA training in mice (Tian et al., 2012). In contrast, a study on rats found that following cocaine self-administration, extinction, and cue reinstatement, there were no changes in Dnmt expression levels or overall DNA methylation in the PFC (Wright et al., 2015). These data point to a more transient role of DNMT in the PFC, where methylation changes are important during the acquisition phase of drug-seeking behavior, but not during the relapse.

Systemic or whole brain pretreatment with Met methionine is also widely used. Wright et al. (2015) subjected a cohort of animals to systemic methionine injections, followed by 10 days of cocaine SA training, and then 10 days of extinction. Although supplemental methionine had no effect on the establishment or extinction of cocaine-seeking behavior, animals who had received methionine injections exhibited less drug seeking behavior during the cocaine-induced reinstatement trial. Interestingly, this reduction in the reinstatement response was not observed in methionine-treated animals that underwent training for sucrose pellet SA. This finding supports the hypothesis that DNA methylation plays a more significant role in motivation-driven behaviors related to stronger, more rewarding stimuli. Wright et al. (2015) found that daily injections of exogenous L-methionine (MET) 1–2 h prior to all operant sessions decrease cocaine priming-induced reinstatement but not SA, extinction, or cue-induced reinstatement. In addition, a study compared plasma oxytocin and blood DNA methylation in the OXTR gene between people with and without cocaine use disorder (CUD). This study found that CUD is associated with higher peripheral oxytocin levels in men during acute abstinence. This finding may be considered in future studies that aim at using exogenous oxytocin as a potential treatment for cocaine addiction (Souza et al., 2023). It is also noted that the cocaine-reinforcing effects are not mediated by DNMT3a2; rather, this enzyme regulates cocaine-cue memories that drive reinstatement to seek cocaine following early abstinence and the incubation of cocaine craving (Cannella et al., 2018).

### 3.4 Intergenerational inheritance

When a father and/or a mother becomes addicted to cocaine, the genetic characteristics of their offspring may also be influenced via DNA methylation, leading to susceptibility to addiction and impaired cognition. The hippocampus of rats exposed to cocaine *in utero* are characterized by altered global patterns of DNA methylation and corresponding changes in gene transcription (Novikova et al., 2008). Specifically, an addicted mother’s DNA methylation patterns may be altered, and these alterations may be passed on to her offspring through reproductive cells (such as egg cells), resulting in differences in gene expression in the offspring. Offsprings of mothers exposed to cocaine exhibited higher DNA methylation and overexpression of DNMTs (Nielsen et al., 2012b). Novikova et al. (2008) found that maternal cocaine exposure in pregnancy alter the methylation patterns in young pups. The study shows an increase in global methylation in the hippocampal pyramid neurons of the young pups at 30 days postnatal. This is accompanied with an attenuated expression of DNMT1 and DNMT3a, while DNMT3b expression remains the same. They also found a decrease in the global DNA methylation in the same region at 3 days postnatum with no change in the expression of DNMTs.

In addition to presenting a significant health burden to adolescent and adult users, prenatal exposure to cocaine is associated with impairments in brain development and cognitive functioning that may last through the school-age years (Lambert and Bauer, 2012). Prenatal cocaine exposure results in increased anxiety-like behavior and impaired spatial learning in male and female offspring into adulthood (Zhao et al., 2015). However, how these changes will affect future drug-using behaviors and related neurobiology remains to be further investigated.

Fathers who are addicted to cocaine can influence the genetic characteristics of their offspring through DNA methylation, much like mothers do. Specifically, the DNA in the sperm of addicted fathers may undergo particular methylation changes that are transmitted to their children during fertilization, leading to variations in gene expression. These changes may affect genes related to addiction, withdrawal responses, and cravings. Rodents self-administering cocaine show decreased DNMT1 in the seminiferous tubules (He et al., 2006), indicating that cocaine may also have DNA methylation effects on the male germ line which could be transmitted to subsequent generations. Cocaine SA produces hypomethylation of Cdkn1a in sperm and a selective increase in the expression of this gene in the NAc of male offspring, which is associated with blunted cocaine reinforcement (Swinford-Jackson et al., 2022). However, various studies involving paternal cocaine administration have demonstrated enhancement in drug susceptibility and related reward-based behaviors in their off springs through epigenetics processes (Vassoler et al., 2013; White et al., 2016; Wimmer et al., 2017). This transgenerational genetic effect may be related to DNA methylation modifications in sperm. DNA methylation patterns in the sperm of addicted fathers may affect the function and connectivity of addiction-related neural pathways in the brains of their offspring, thereby increasing the risk of addiction in their offspring. Additionally, Paternal voluntary administration of cocaine has demonstrated to cause epigenetic germ line reprogramming with increased mRNA and protein level of Brain-derived neurotrophic factor (BDNF), which was also associated with H3 acetylation at BDNF promoters, in medial PFC of only male offsprings (Vassoler et al., 2013). Similar study with paternal cocaine exposure also indicated hippocampal epigenetic remodeling resulting in NMDA receptor–dependent memory formation and impairments in synaptic plasticity of male offsprings (Wimmer et al., 2017).

The intergenerational effects of cocaine use show that methylation patterns may be transmitted through the preservation mechanisms of gametes, including sperm and eggs. Epigenetic alterations could be transmitted to subsequent generations, in parallel with transgenerational inheritance of high responding to drug reward. Active cocaine-seeking behavior in paternal rats, rather than cocaine intake alone, induces specific changes in the DNA methylation patterns of their sperm. These changes are preserved in the sperm of their offspring (F1) and are linked to alterations in gene expression within addiction-related signaling pathways in the NAc. Specifically, highly motivated cocaine-seeking experiences may create “addiction-like behavioral signatures” through DNA methylation (Le et al., 2017). These modifications may evade the usual genomic reprogramming that occurs during spermatogenesis, allowing them to be selectively retained throughout the process of gametogenesis. Although the contribution of sperm DNA methylation to transcriptomic changes of addiction-related signaling pathways in offspring NAc is not clear, the authors speculate that so-called epigenetic engrams of addiction like behavioral experience in previous generations may induce adaptation of brain functions in offspring to facilitate prompt and favorable adaptive responses on their exposure to cocaine. However, although the available evidence supports the crucial role of DNA methylation in transgenerational transmission, the specific mechanisms remain to be explored (Riyahi et al., 2024).

### 3.5 Cell specificity

The above-mentioned cocaine related alteration in DNA methylation is cell-specific, meaning that different cell types differ in their DNA methylation patterns. In a landmark study, Heiman et al. (2008) used translating ribosome affinity purification to isolate and transcriptionally profile dopamine receptor class 1 (D1) and class 2 (D2) expressing MSNs from the striatum of cocaine-exposed BAC transgenic mice. After chronic cocaine injections, Dnmt3a transcription was specifically induced in D1-MSNs and was accompanied by increased GABAergic activity of these cells in response to cocaine in culture (Heiman et al., 2008). These results emphasize the importance of cell-type specificity, and suggest that the increased methyltransferase expression seen in previous studies of striatal tissue may be occurring in specific subtypes of cells.

Although most methylation studies have been on changes occurring in bulk striatal tissue, there has been a small focus on glial cell specific alterations. Nielsen et al. (2012a) trained rats to self-administer cocaine for 14 days and then examined methylation changes at promoters of white matter-related genes in the corpus callosum. Although they investigated three oligodendrocyte specific genes, including the myelin-integrity-related proteins Mbp and Plp1, they only found differential methylation at the promoter of the Sox10 gene, which was significantly less methylated in the cocaine-trained animals after one and 30 days of forced abstinence (Nielsen et al., 2012a). This cell-specific alteration in DNA methylation may be closely related to the pathogenesis of cocaine addiction in humans. Glial cells are an understudied population of cells to investigate cocaine epigenetics, despite their implication in human cocaine-related transcriptional dysregulation (Albertson et al., 2004; Kristiansen et al., 2009). In immortalized microglia from mice, even brief (3 h) exposure to cocaine *in vitro* is followed by a lasting increase in Dnmt1 and Dnmt3a protein expression (Guo et al., 2016). Similarly, moderate doses of cocaine induce Dnmt1 protein expression in rat primary microglia, and the levels of all three classical Dnmts are increased in the microglia of mice after chronic cocaine injections. Guo et al. (2016) used microglial cell lines and FACS sorted microglia from mice to investigate methylation changes at a microRNA gene. Specifically, mMiR-124 may be involved in suppressing microglial activation in response to neuro inflammation (Veremeyko et al., 2013) and is up-regulated in microglia in response to cocaine exposure (Guo et al., 2016). This expression change is accompanied by large increases in methylation of the pri-miR-124a-1 and pri-miR-124-2 gene promoters and supports the theory that DNA methylation plays a regulatory role over other, post-transcriptional regulators, in response to chronic cocaine.

Similar to DNA methylation, the process of demethylation may also be cell-specific. Different cell types differ in the expression and activity of TET, which may result in different sensitivities to the cocaine withdrawal response. A study that employed targeted next-generation bisulfite sequencing (TNGBS) on human astrocytes exposed to cocaine revealed changes in the levels of DNMT1, DNMT3a, DNMT3b, TET1, TET2, and TET3, as well as a reduction in mitochondrial DNA (mtDNA) methylation levels (Doke et al., 2021).

The expression of Mecp2 also suggest cell-specific DNA methylation and demethylation. In animals given extended access (6 h per day) to a cocaine-associated lever, the number of Mecp2 positive cells in the dorsal striatum was significantly increased. But Mecp2 positive cells tended to co-localize with the neuronal marker NeuN, and were not increased in animals given limited (1 h per day) access to drug SA. These results suggest that cocaine SA, particularly over an extended time period or with a higher cumulative dosage, induces the expression of Mecp2 and the activation of related neurotrophic and transcriptional pathways in the neurons in the dorsal striatum (Im et al., 2010).

## 4 Opiates and epigenetic modification of DNA

Opioid addiction is a large category of drug use disorders, such as heroin, morphine, and oxycodone, which are usually administered intravenously as painkillers and can cause physical dependence and tolerance, and the addiction mechanism is closely related to DNA methylation.

### 4.1 Drug exposure

There has been little study has revealed the associations between acute opioid exposure and DNA methylation. In a genome-wide DNA methylation study of saliva samples from opioid-naive dental surgery patients, nine out of ten selected CpG sites in the opioid receptor μ-1 (OPRM-1) promoter show elevated methylation in patients approximately 40 days after the surgery following higher doses of morphine treatment (Sandoval-Sierra et al., 2020), suggesting that human OPRM1 promoter is hypermethylated following acute exposure to opiates.

The effects of chronic opioids on DNA methylation are more complex. Most studies focus on OPRM-1 gene, because bioactive products of opioid, such as morphine, β-endorphin and opioid analgesic medications, mainly target the OPRM-1 (Kreek et al., 2012). Post-mortem brain samples from long-term opioid abusers have been reported to exhibit reduced OPRM1 expression that was related to altered methylation at a CpG site in the functional SNP variant of the OPRM1 (118A > G) gene (Oertel et al., 2012). Furthermore, OPRM1 protein level in the frontal cortex of patients who died of a heroin or methadone overdose was not different to the level observed in control subjects (García-Sevilla et al., 1997). However, another study found decreased level of this receptor in the PFC (Ferrer-Alcón et al., 2004). Recent study found that the patients with opioid use disorder (OUD) showed a significant decrease in OPRM1 DNA methylation (CpG sites 1-5 selected in the promoter region) in long opioid-treated chronic non-cancer pain, which showed significant gender difference. Significant differences were found at the five CpG sites studied for men, and exclusively in women for CpG site 3, in relation to OUD diagnosis (Agulló et al., 2024). The data collectively indicate that opioids can stimulate DNA methylation of the OPRM1 gene. These different findings, which were probably due to the different drug types, drug treatment patterns, and testing tissues or cells, suggest that cell specific modification target DNA methylation is necessary to future clinical use.

In addition to opioid receptor genes, OUD has been associated with DNA methylation of other genes. Although opioid dependence is more prevalent in men, opioid relapse and fatal opioid overdoses have recently increased at a higher rate among women. The first epigenome-wide association study (EWAS) of opioid dependence in European-American (EA) women identified three genome-wide significant differentially methylated CpG sites mapping to the PARG, RERE, and CFAP77 genes. These genes are involved in chromatin remodeling, DNA binding, cell survival, and cell projection (Montalvo-Ortiz et al., 2019).

Genome-wide DNA methylation appears to be elevated by long-term heroin use based on higher levels of methylation at LINE-1 retrotransposon sites in blood leukocytes from heroin addicts compared with control subjects (Doehring et al., 2013). An epigenome-wide analysis of DNA methylation in brain samples of individuals who died from acute opioid intoxication and group-matched controls showed that axonal growth-inducing factor Netrin-1 in the dorsolateral PFC (dlPFC) may be associated with opioid overdose (Shu et al., 2021).

Serotonin (5-HT) is implicated in the reward processes underlying SUD. A study (Li et al., 2022) determined the associations between several single-nucleotide polymorphism (SNPs) in three representative 5-HT receptor genes (HTR1B, HTR2A, and HTR3B) and susceptibility to heroin use disorder. rs6296 in the 5-Hydroxytryptamine Receptor 1B (HTR1B) gene was correlated with susceptibility to heroin use disorder. The CpG sites HTR1B_07 and HTR1B_26 and the promoter region of the HTR1B gene were hypermethylated in patients with heroin use disorder. Notably, rs6296 correlated in an allele-specific manner with methylation in the HTR1B gene promoter in the blood and gene expression of the HTR1B gene in the frontal cortex and hypothalamus. Collectively, SNP rs6296 is associated with OUD by involving mechanisms of DNA methylation and expression of the HTR1B gene.

Because of its importance in opiate-induced plasticity and reward activity, the expression level and DNA methylation of BDNF have been widely studied. A study showed a higher methylation and therefore a 35% reduction of BDNF serum levels in patients taking methadone as maintenance treatment when compared with healthy volunteers or patients with medicalized heroin (Schuster et al., 2017). Another study found that repeated morphine administration induces selective demethylation of Bdnf exon IV promoter and increase of BDNF expression in dorsal root ganglion neurons (Chao et al., 2016).

Although chronic morphine or heroin administration with stable or escalating doses did not alter whole-brain DNA methylation (Fragou et al., 2013; Chao et al., 2014). One study identified several changes to global or promoter-specific 5mC and 5hmC levels across multiple brain regions following chronic morphine exposure in rats (Kenny, 2014). Whether these changes have functional consequences at the level of gene expression or behavior remains to be determined. In conclusion, opioid exposure can affect DNA methylation levels, and this effect can be realized through both opioid receptor pathways and non-opioid receptor pathways.

### 4.2 Addiction related memory

**FIGURE 1:**
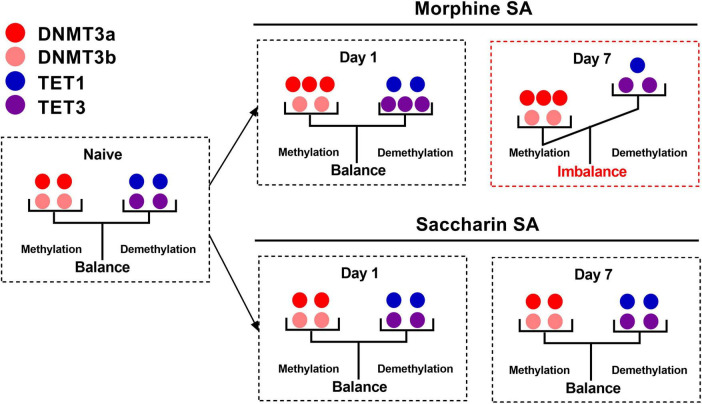
Diagram of the balance between DNA methylation and DNA demethylation after 1- or 7-day morphine self-administration (SA) training/saccharin SA training. In CA1, 1-day morphine SA training up-regulated the expression of TET3 and DNMT3a, resulting in a balance between DNA methylation and DNA demethylation, but 7-day morphine SA training up-regulated the expression of DNMT3a and down-regulated the expression of TET1 and TET3, resulting in an imbalance between DNA methylation and DNA demethylation. Saccharin SA had no effect on this balance. DNMT3a, DNA methyltransferase 3a; DNMT3b, DNA methyltransferase 3b; TET1, ten-eleven translocation 1; TET3, ten-eleven translocation 3.

DNA methylation and demethylation in brain regions such as hippocampal CA1 and amygdala plays an important role in opioid addiction related memory. In the SA model, animals obtain opioids through SA behavior and gradually develop drug dependence and addiction memory. A previous study from our lab has demonstrated a key role for DNMT3a in the acquisition of morphine SA in rats. The expression of DNMT3a in the CA1 but not in the NAc shell was significantly up-regulated after 1- and 7-day morphine SA but not after the yoked morphine injection. Meantime, saccharin SA did not affect the expression of DNMT3a or DNMT3b. DNMT inhibitor 5-aza or RG108 microinjected into the hippocampal CA1 or knockdown of DNMT3a significantly attenuated the acquisition of morphine SA (Liu et al., 2016). We also showed that 1 day of morphine SA training upregulated TET3 but not TET1 expression in the hippocampal CA1. With 7 days of morphine SA training, the expression of TET3 in the CA1 returned to the baseline level, while the TET1 expression was downregulated. No change of TET1 and TET3 in the NAc shell was observed in morphine SA trained rats, or in the yoked morphine rats, or in rats trained for saccharin SA. Furthermore, we found that knocking down TET3 expression in the CA1 accelerated the acquisition of morphine SA, while overexpression of the catalytic domain of TET1 in the CA1 attenuated the acquisition (Zhang et al., 2020). So DNMT may inhibit the expression of certain genes by promoting DNA methylation, while TET enzymes may activate the expression of these genes by demethylation. This mutually antagonistic mechanism of action may play an important role in opioid use disorder. Together, these findings suggest that the balance between DNA methylation and demethylation, rather than DNA methylation itself, in the CA1 are important epigenetic modulators involved in the morphine-seeking behavior and provide a new strategy in the treatment of opioid addiction (Figure 1).

Another study in our lab found that DNA hypermethylation of Gnas in the BLA but not in the NAc is necessary for the reconsolidation of morphine reward memories (Liu et al., 2022). Furthermore, Neither morphine treatment nor heroin SA altered DNA methylation in the mesocorticolimbic dopamine system of rodents (Bourtchouladze et al., 2006; MacDonald et al., 2007), which contrasts with evidence that cocaine alters global DNA methylation in the PFC and NAc (Auber et al., 2013).

Interestingly, DNMT3a is degraded by the E2 ubiquitin-conjugating enzyme Ube2b-mediated ubiquitination in dorsal hippocampus of rats that repeatedly self-administrated heroin. DNMT3a degradation leads to demethylation in calcium/calmodulin-dependent kinase kinase 1 (CAMKK1) gene promotor, thereby facilitating CaMKK1 expression and consequent activation of its downstream target CaMKIα. CaMKK1/CaMKIα signaling regulates actin cytoskeleton remodeling in the dorsal hippocampus and behavioral plasticity by activation of Rac1 via acting Rac guanine-nucleotide-exchange factor βPIX. These data suggest that Ube2b-dependent degradation of DNMT3a relieves a transcriptional brake on CaMKK1 gene and thus activates CaMKK1/CaMKIα/βPIX/Rac1 cascade, leading to drug use-induced actin polymerization and behavior plasticity (Chen et al., 2021; Jiang et al., 2021).

Conditioned positional favoritism is another commonly used model for studying addictive reward memory. Injection of a 5-aza into CA1 significantly attenuated morphine-induced CPP consolidation, acquisition, and recovery in rats, and inhibition of DNA methylation in the PL region enhanced mCPP retrieval. All these behavioral effects were absent when OA was infused before 5-aza injection. These findings suggest that 5-aza interfere opiate-related memory, and protein phosphatase plays an important role in this process (Zhang et al., 2014). In another study, using the morphine-naloxone induced conditioned place aversion (CPA) model in rats, we injected 5-aza into agranular insular (AI), granular insular (GI), basolateral amygdala (BLA) and central amygdala (CeA) immediately after the memory retrieval. We found that 5-aza injection into AI and BLA but not into GI or CeA attenuated or disrupted the reconsolidation of morphine-associated withdrawal memory.

In addition, a study explored the mechanism of Oxycodone (Oxy) addiction and the role of Oxytocin (OT) in Oxy-induced epigenetic alterations. Oxy chronic exposure induced DNA hypomethylation at the exons of the Arc, Dlg1, Dlg4, and Syn1 genes. It also down-regulated DNMT1 and up-regulated TET1-3, leading to a decrease in global 5-mC levels and differential demethylation at exon 1 of Synaptophysin (Syn) and exon 2 of post-synaptic density protein 95 (Psd95). These changes elevated the expression of synaptic proteins (SYN, PSD95) and synaptic density in the VTA. Moreover, pretreatment with OT (i.c.v.) blocked Oxy CPP, normalized synaptic density, and regulated DNMT1 and TET2-3 causing reverse of DNA demethylation of Syn and Psd95 (Fan et al., 2019). Intracerebroventricular (ICV) administration of oxytocin specifically blocked oxycodone relapse, possibly by inhibition of Arc, Dlg1, Dlg4, and Syn1 hypomethylation in oxycodone-treated rats. Together, these data indicate the occurrence of epigenetic changes in the hippocampus following oxycodone relapse and the potential role of oxytocin in oxycodone addiction (Fan et al., 2021). In summary, there is a close relationship between opioid use disorders in terms of addiction memories and DNA methylation, which may modulate the formation, consolidation and reconsolidation of addiction memories.

### 4.3 Withdrawal and relapse

Opioid withdrawal induced neuroadaptive changes may lead to alterations in DNA methylation status, which further affect gene expression, thereby exacerbating the onset of withdrawal symptoms and increasing the risk of relapse. For instance, morphine treatment of neonatal abstinence syndrome (NAS) is associated with decreased DNA methylation at 1 of 4 CpG sites within the OPRM1 gene (Camerota et al., 2022). Chronic morphine exposure induces alterations in the redox state of neuron and decreases in DNA methylation levels, and the combined use of D-cysteine ethyl ester (D-CYSee) and betaine is effective in attenuating the morphine withdrawal response in rats (McDonough et al., 2024). Oxycodone relapse was also related to markedly decreased 5-mC levels and decreased transcription of Dnmt1, Dnmt3a, and Dnmt3b; in contrast, 5-hmC levels and the transcription of Tet1 and Tet3 were increased (Fan et al., 2021). Therefore, understanding the altered DNA methylation status during opioid withdrawal and its relationship with gene expression is critical to unraveling the neurobiological mechanisms of drug addiction, predicting the severity of withdrawal symptoms, and developing effective intervention strategies.

### 4.4 Intergenerational inheritance

Opioid abuse during pregnancy may result in Neonatal Opioid Withdrawal Syndrome (NOWS). Children with NOWS have abnormal DNA methylation patterns. A genome-wide methylation analyses of 96 placental tissue samples provides strong association between DNA methylation dysregulation of placental tissue and NOWS development (Radhakrishna et al., 2021). Another study assessed relationships between placental DNA methylation with *in utero* opioid exposure and NOWS severity. They found that lower placental ABCB1 methylation was associated with severe NOWS. Higher placental CYP19A1 methylation correlated with higher umbilical cord norbuprenorphine levels (Townsel et al., 2024). Disruption of DNA methylation patterns in CYP genes play a role in NOWS and serve as a biomarker for future avenues of personalized therapy. Cytochrome P450 (CYP) enzymes play a pivotal role in metabolizing a wide range of substances in the human body, including opioids, other drugs, toxins, and endogenous compounds. The study identified 20 significantly differentially methylated CpG sites associated with 17 distinct CYP genes, with 14 CpGs showing reduced methylation across 14 CYP genes, while 8 exhibited hypermethylation, and 5 genes exhibited both increased and decreased methylation. These genes are crucial for metabolizing eicosanoids, fatty acids, drugs, and diverse substances. This suggests that disruption of DNA methylation patterns in CYP transcripts might play a role in NOWS and may serve as valuable biomarkers, suggesting a future pathway for personalized treatment. Further research is needed to confirm these findings and explore their potential for diagnosis and treatment (Radhakrishna et al., 2024).

A pilot study comparing cord blood samples from full-term infants of methadone maintenance treatment opioid-dependent (MMOD) mothers with those of untreated opioid naïve mothers analyzed genome-wide DNA methylation differences. A total of 152 differentially methylated sites were identified, of which 90 were hypermethylated (involving 59 annotated genes) and 62 were hypomethylated (involving 38 annotated genes). These DNA methylation changes involved multiple genes, pathways, and networks, including in the areas of cell growth, neural development, vision, and xenobiotic metabolic functions. Furthermore, following opioid exposure, hypermethylation within the OPRM1 promoter is repeatedly observed in infant’s cord blood or saliva with neonatal abstinence syndrome, suggesting the likelihood of OPRM1 gene suppression in the offspring (Wachman et al., 2014). Interestingly, elevated DNA methylation at the OPRM1 promoter is also reported in the spermatozoa of humans with a history of opioid use, suggesting an intergenerational epigenetic inheritance of chronic opioid-induced DNA hypermethylation (Chorbov et al., 2011). Hence, both parental and in- utero opioid exposures affects the methylation patterns in offspring, which may influence their overall drug sensitivity and behavior and can lead to predisposition to addiction (Trivedi et al., 2014).

### 4.5 Cell specificity

Cell-specific DNA methylation plays a crucial role in modulating cell functions and is often disrupted in SUD. In sensory neurons, cell and exon-specific DNA hypomethylation permits CTCF binding is the master regulator of mammalian chromatin structure, this regulation subsequently influences cell-specific alternative splicing of the presynaptic calcium channel gene, Cacna1b, impacting opioid sensitivity (Cunha-Oliveira et al., 2013). Another study generated genome-wide transcriptomic and DNA methylation profiles of OUD subjects and non-psychiatric controls and found that the DNA methylation in astrocyte and glial cell may involve in OUD. They found 6 OUD-associated co-expression gene modules and 6 co-methylation modules. Genes in these modules are involved in astrocyte and glial cell differentiation, gliogenesis, response to organic substance, and response to cytokine (Liu et al., 2021).

The alteration in DNA methylation is observed in both intracerebral (brain) and peripheral tissues. In the peripheral tissue, patients with a prior history of heroin use and that were on methadone maintenance treatment show increased methylation at two CpG-rich islands in peripheral lymphocytes. The methylated sites are located at the predicted Sp1 transcription factor-binding sites and may prevent Sp1 and other transcriptional activators from accessing the locus, resulting in a low expression of OPRM1 as noted in the lymphocytes of these patients (Nielsen et al., 2009). Genomic DNA was used for analysis of DNA methylation in the region 1 (R1) and region 2 (R2) of the OPRM-1 promoter. The leukocytes of the group comprising males with opium use disorder demonstrated hypermethylation in the R2 of the OPRM-1 promoter, which showed that altered DNA methylation status increase the risk of opioid abuse in patients (Ebrahimi et al., 2018). Similarly, increased DNA methylation is also reported at an additional CpG-rich island of the OPRM1 promoter in leukocytes of prior opiate users; but DNA methylation at this site does not induce a change in the transcriptional level of OPRM1 (Doehring et al., 2013).

## 5 Methamphetamine and epigenetic modification of DNA

Methamphetamine (METH) is an addictive psychostimulant drug that acts on the central nervous system (CNS). It is commonly used as a recreational drug and sometimes to treat obesity and attention-deficit hyperactivity disorder. METH is one of the most widely distributed psychostimulants worldwide. Despite active counter measures taken by different countries, neither overall usage of METH nor the frequency of repeat users has reduced over the past decade. METH induces abuse and dependence as it acts on the central nervous system and temporarily stimulates the brain. The recidivism rate for abuse of stimulants is very high and therefore prevention of repeated usage is paramount (Yuka et al., 2020).

### 5.1 Drug exposure

Long-term METH drug use produces adaptive changes in the central nervous system, including imbalances in neurotransmitter systems and abnormalities in reward circuits. These alterations are often closely associated with persistent changes in DNA methylation levels. Fisher 344 rats exhibited increased striatal DNMT1 expression after both acute and repeated injections of METH, whereas Lewis rats showed reduced DNMT1 expression. Interestingly, they also reported increased DNMT1 expression in the NAc of Fisher rats but not in the Lewis rats (Numachi et al., 2007). Moreover, METH increased the expression of the DNMT1 (Numachi et al., 2007; Jayanthi et al., 2014).

A study showed that following the binge METH treatment in rats, methylation of CpG-2 significantly decreased, whereas chronic METH treatment resulted in hypermethylation of the same site (Moszczynska et al., 2017). Chronic METH exposure may also lead to stable changes in DNA methylation patterns, so-called “epigenetic imprints”, which may persist after withdrawal and affect an individual’s behavioral and cognitive functioning. Enzyme-linked immunosorbent assay (ELISA)—based DNA methylation determination found increased 5-mC levels in the pCTX after chronic METH exposure (González et al., 2018). In addition, detection of DNA methylation levels by methylight qPCR in male METH addicts revealed significant correlation between the methylation levels of chimerin 2 (CHN2) and METH dependence (Hao et al., 2017). CHN2 is a protein involved in remodeling of the actin cytoskeleton and hippocampal axonal pruning (Riccomagno et al., 2012). This suggests that prolonged abuse of METH induces abnormal methylation of CHN2 gene that interferes in actin skeleton remodeling leading to irregular formation of neurites and growth cones that is considered crucial to maintaining long-lasting addictive behaviors (Nestler, 2013). Interestingly, a research group (Yuka et al., 2020) has recently identified the gene Shati/Nat8L as a medical marker for MUD diagnosis according to pre-clinical studies (Niwa et al., 2007; Uno et al., 2017; Haddar et al., 2020). This research reported that the ratio of DNA methylation in SHATI/NAT8L was significantly higher at six CpG sites in METH users when compared to healthy subjects (Yuka et al., 2020).

BDNF plays key roles in neuronal protection and synaptic plasticity. Changes in BDNF are associated with various pathological conditions, including METH addiction and is implicated in psychiatric conditions reminiscent of those suffered by METH-abusers. PFC is involved in METH addiction and in mental disorders similar to those triggered by METH. A study has shown that unbalanced BDNF level and methylation of its CpG island in the PFC may have crucial roles in the complications of METH abuse (Salehzadeh et al., 2020). BDNF methylation is abnormal in human METH dependence, especially METH-dependent psychosis, and in METH-administered rats. This may influence BDNF expression and contribute to the neurotoxic effects of METH exposure (Iamjan et al., 2021). Hippocampal CA1 BDNF protein was significantly increased in the escalating dose (ED)-binge rats, while other hippocampal regions and frontal cortex were not significantly affected. Meth-administered animals also demonstrated deficits in Novel object recognition (NOR) after 24 h delay. No significant effect of the additional binge dose on BDNF protein or NOR findings was apparent. The hippocampal BDNF increase may reflect an initial increase in a protective factor produced in response to elevated glutamate release resulting in neurodegenerative excitotoxicity (Iamjan et al., 2024).

Chronic METH exposure may also affect the DNA demethylation, leading to aberrant maintenance of the DNA methylation state. A study found that METH-induced increased TET1 and TET3 levels in the NAc. METH increased TET1 binding at the corticotrophin releasing hormone (Crh) promoter and increased TET3 binding at vasopressin (Avp) intragenic regions, the TET inhibitor, 1,5-isoquinolinediol (IQD), blocked the change of Crh and Avp mRNA levels (Jayanthi et al., 2018), these suggested TET-induced DNA hydroxymethylation in the NAc is an important driver of the effects of METH. In contrast, increasing doses of METH over a period of 2 weeks led to decreased enrichment of 5-hmC at the promoter regions of striatal AMPA glutamate receptors in rats (Jayanthi et al., 2014). Methylated DNA immunoprecipitation and hydroxymethylated DNA immunoprecipitation-polymerase chain reaction also revealed that chronic METH was associated with decreased enrichment of 5-mC and 5-hmC at GluA1 and GluA2 promoter sequences. These results indicate that comparable changes in DNA methylation and hydroxymethylation can occur in METH use disorder. The relevance of these changes in behavioral responses to the drug will require specific genetic manipulations of the enzymes involved in catalyzing each reaction in the brain.

In response to the DNA methylation and demethylation mechanisms of METH addiction, researchers are also exploring a variety of interventional treatment strategies to enhance or inhibit the effects of these mechanisms. For example, some drugs may be able to inhibit the activity of DNMTs, thereby decreasing the level of DNA methylation and thus reversing the gene expression alterations associated with METH addiction. Other drugs may restore normal gene expression by facilitating the demethylation process. A research team tested the role of TET enzymes in METH-induced changes in gene expression by using the TET inhibitor, 1,5-isoquinolinediol (IQD), and found that IQD blocked METH-induced increases in Crh and Avp mRNA expression (Lukowiak et al., 2014).

### 5.2 Addiction related memory

Cadet et al. (2019) have found that METH pretreatment enhances METH SA, which is accompanied by increased DNA methylation at the DNA sequences of several potassium channels genes. METH pretreatment caused enhancement of escalated METH SA and down-regulated mRNA and protein expression of voltage-gated K + channels (Kcna1, Kcna3, and Kcnn1). The epigenetic mechanisms underlying the transcriptional alterations observed may be due to increased DNA methylation at the CpG-rich sites on their promoter sequences (Jayanthi et al., 2020).

Oxytocin(OT)plays a central role in learning and memory, but little is known of the impact of OT on METH-induced abnormal memory. However, it has been suggested that OT is involved in the formation and maintenance of addiction memories by influencing neuronal activity and synaptic plasticity. These processes may interact with DNA methylation and demethylation mechanisms to co-regulate addiction memory. Fan et al. (2020) found the role of OT in METH-induced epigenetic alterations that underlie spatial and cognitive memory changes. METH enhanced spatial memory, decreased synapse length, downregulated DNMT1, DNMT3A, DNMT3B, and MECP2, and induced DNA hypomethylation at the Syn promoter in Hip. In contrast, METH reduced cognitive memory, increased synapse thickness, upregulated DNMT1, DNMT3A, and MECP2, and induced DNA hypermethylation at the Syn promoter in PFC. OT pretreatment specifically ameliorated METH-induced learning and memory alterations, normalized synapse structures, and regulated DNMTs and MECP2 to reverse the DNA methylation status changes at the Syn promoter in Hip and PFC. So, DNA methylation may be an important gene regulatory mechanism underlying METH-induced learning and memory alterations. OT can potentially be used to specifically manipulate METH-related memory changes (Fan et al., 2020).

### 5.3 Withdrawal and relapse

The DNA methylation and demethylation in METH withdrawal and relapse involves the NAc, striatum, and interpeduncular nucleus. Altered DNA methylation status in these brain regions may affect the expression of addiction-related genes and neuronal activity, thereby modulating addictive behaviors and the tendency to relapse after withdrawal. Studies have shown that METH addiction is associated with changes in DNA methylation and demethylation of potassium ion channels in the NAc. Because compulsive METH takers and METH-abstinent rats show differences in potassium (K^+^) channel mRNA levels in their NAc, A study explored that if K^+^ channel expression might also help to distinguish between a single saline injection followed by METH SA (SM) and a single METH injection followed by METH SA (MM) groups. The authors found increases in mRNA and protein expression of shaker-related voltage-gated K^+^ channels (Kv1: Kcna1, Kcna3, and Kcna6) and calcium-activated K^+^ channels (Kcnn1) in the SM compared to MM rats, SM rats also showed decreased DNA methylation at the CpG-rich sites near the promoter region of Kcna1, Kcna3 and Kcnn1 genes than the other MM rats (Jayanthi et al., 2020).

In order to further test the role of DNA hydroxymethylation in METH use disorder, Cadet et al. (2017) distinguish rats into non-addicted from addicted rats in the presence of adverse consequences represented by contingent foot shocks. They reported that there were differentially hydroxy methylated regions in genes encoding voltage (Kv1.1, Kv1.2, Kvb1, and Kv2.2)- and calcium (Kcnma1, Kcnn1, and Kcnn2)-gated potassium channels observed in the NAc of non-addicted rats, with these changes being associated with increased mRNA levels of these potassium channels in comparison to compulsive and control rats (Cadet et al., 2017). Thus, changes in differentially hydroxymethylated regions and increased expression of specific potassium channels in the brain may suppress METH-taking behaviors in the presence of adverse consequences. These observations support the idea of using potassium channel activators to treat METH use disorder in humans (McCoy et al., 2021).

The striatum is an important region of the brain associated with motor control and reward systems. In METH addiction, the DNA methylation status of the striatum may also be altered. DNA methylation regulates the potential for astrocyte to neuronal differentiation in striatal brain regions, which may be related to changes in neuroplasticity following METH addiction. In addition, altered DNA methylation status in the striatum may also affect the functioning of the reward system, thereby modulating addictive behaviors and the tendency to relapse after withdrawal. A study analyzes the persistence of Meth-induced striatal synucleinopathy at a prolonged time interval of Meth withdrawal found that exposure to high and/or prolonged doses of METH, apart from producing nigro-striatal toxicity, determines a long-lasting increase in striatal alpha-synuclein levels. Moreover, the persistently demethylation within alpha-synuclein gene (SNCA) promoter matches the increase in alpha-synuclein protein. The demethylation was remarkable (ten-fold of controls) and steady, even at prolonged time intervals being tested so far (up to 21 days of METH withdrawal). Similarly alpha-synuclein protein assayed stoichiometrically steadily increased roughly ten-fold of controls. Therefore, METH persistently increases alpha-synuclein and suppresses gene promoter methylation within striatal neurons (Biagioni et al., 2019).

### 5.4 Intergenerational inheritance

One of the significant METH-related public health consequences concerns the long-term effects of METH on brain development and associated behaviors in children born to addicted mothers. There are several findings that show multi-generational effects in the offspring of pregnant rodents exposed to METH during the gestational period. Prenatal METH exposure in a rat model demonstrated METH-induced behavioral sensitization and NAc DNA methylation changes in male offspring. Altered DNA methylation in the NAc was associated with 86 annotated genes functionally enriched in the pathways of neurodevelopment and addiction, including Kirrel3, Lappic and Peg3 (Dong et al., 2022). A longitudinal study in children with *in utero* exposure to METH found increased DNA methylation at the CpG2 site of HSD11B2, a stress-related gene (Oni-Orisan et al., 2021). METH-addicted mothers may pass the drug or its metabolites to the fetus or infant through the placenta or breast milk, thereby affecting their DNA methylation patterns. On the other hand, the genetic characteristics of the father and environmental factors may also influence the gene expression of the child through DNA methylation patterns in the sperm. However, the influence of fathers on the DNA methylation patterns of their children may be more complex and indirect than that of mothers. In a recent study, the METH exposure obviously altered F0 sperms DNA methylated profile and male F1 mPFC transcriptomic profile, many of which being related to neuronal system and brain development. In METH-sired male F1, subthreshold dose of METH administration effectively elicited CPP, along with more mPFC activation. After qPCR verification, Sort1 and Shank2 were at higher levels in F0 sperm and F1 mPFC. Their findings put new insights into paternal METH exposure-altered profiles of F0 sperm DNA methylation and male F1 mPFC transcriptomics. Sort1 and Shank2 might be used as potential molecules for further research on the transgenerational vulnerability to drug addiction in offspring by paternal drug exposure (Li et al., 2024).

Genetic traits of both parents and environmental factors combine to influence the DNA methylation patterns of their children. In rodent studies, several lines of evidence suggest differences in DNA methylation in the hippocampus of the offspring of male and female mice exposed to MA. Itzhak et al. (2015) exposed male and female mice to increasing doses of METH right from adolescence to adulthood, and the female mice were continuously given METH through the gestation. They found that the offspring showed METH induced differentially methylated regions (DMRs) throughout the hippocampal region (Itzhak et al., 2015). Interestingly, hippocampal DNA methylation studies revealed that there were significant differentially methylated regions consequent to *in utero* METH exposure. These data are consistent with the idea that METH-taking during pregnancy may cause substantial alterations in epigenetic markers during brain development (Salinas et al., 2020; Vissers et al., 2020). The authors showed male offspring with prenatal parental METH exposure had increased cocaine-induced conditioned place preference testing and hyperactivity. Hippocampal DNA methylation analysis focused on differentially methylated promoter regions and identified 62 elevated and 35 reduced promoter regions with DNA methylation following prenatal METH exposure (Itzhak et al., 2015). So, the deleterious effects of METH exposure extend beyond abusers, and may potentially impact the vulnerability of their offspring in developing addictive behaviors.

### 5.5 Cell specificity

Of particular interest in exploring the mechanism of action of DNA methylation in METH addiction is its specific effects in different cell types. In one study, a positive impact intervention implemented for a targeted minority of men with AIDS, aimed at changing their METH-using behavior, observed significant changes in leukocyte DNA methylation, suggesting that DNA methylation may be an important biological mechanism by which interventions influence drug abuse behavior (Carrico et al., 2024). Of particular importance, METH has also been found to consistently increase the level of α-synuclein in striatal neurons and inhibit the methylation of the promoters of related genes (Biagioni et al., 2019), which not only reveals the direct impairment of neuronal function by the drug, but also underscores the central role of DNA methylation in neuronal cell-specific responses. Thus, the mechanism of DNA methylation action in METH addiction exhibits complex and specific patterns in different cell types, providing an important perspective for understanding the biological basis of drug addiction and developing effective intervention strategies.

In METH addiction, the DNA methylation of the interpeduncular nucleus may also be altered. Long-term METH withdrawal induces anxiety-like behaviors accompanied by hyperactivation of gamma-aminobutyric acid (GABA)-ergic neurons in the interpeduncular nucleus, and that this activation may be related to DNA methylation or demethylation. The parvalbumin (PV)-containing subgroup of GABAergic neurons is particularly affected in schizophrenia and METH-induced psychosis. There is one study investigated whether METH dependence and psychosis may involve an effect on DNA methylation of the parvalbumin (PVALB) promoter. A significant increase in PVALB methylation was observed in METH dependence and METH-induced psychosis. These results demonstrate a specific association between elevated PVALB methylation and METH-induced psychosis. This finding may contribute to the GABAergic deficits associated with METH dependence (Veerasakul et al., 2017).

## 6 Discussion

Epigenetic modifications in response to drugs of abuse is emerging as a promising field in studying the alteration of DNA methylation patterns in specific gene promoter sites as well as the whole genome. There is a growing body of evidence suggesting the significance of DNA methylation in drug dependence. Our partial answer in this paper to the effect of drug abuse on methylation levels and regulation of gene expression of specific genes at different aspects contributes to a further understanding of epigenetic mechanisms, thus opening up new areas for therapeutic and investigative research.

The DNA methylation that occurs in specific brain regions and cell types can affect the function of these brain regions or cells by regulating the expression levels of crucial genes, ultimately leading to changes in addictive behavior. Cocaine-primed reinstatement induced upregulation of the immediate early gene c-Fos in the NAc and mPFC and reduced methylation at CpG dinucleotides in the c-Fos gene promoter (Wright et al., 2015), suggesting that drug-seeking behaviors are, in part, attributable to a DNA methylation-dependent neuronal activation process, likely occurring at specific gene loci (e.g., c-Fos) in the reward pathway. The DNA methylation can also mediate addictive behavior via an indirect way. Previous studies have demonstrated that cocaine exposure increased DNA methylation of miR-124 promoter, resulting into decreased microglial miR-124 levels as well as microglial activation, leading to the production and release of pro-inflammatory factors, including TNFa, IL-6, and CCL2 (Yao et al., 2010; Guo et al., 2015; Liao et al., 2016). On the other hand, pro-inflammatory cytokines have been demonstrated to be involved in drug addiction. For example, methamphetamine increased the mRNA levels of IL-6 and TNFa in the striatum and hippocampus (Gonçalves et al., 2008; Wisor et al., 2011), and TLR4 knockout mice showed decreased cocaine-induced CPP and SA (Northcutt et al., 2015). These findings suggest that the DNA methylation in microglia may mediate SUD via microglial activation, which add further validity to the SUD-mediated neuroinflammation theory.

Neuronal ensembles may exemplify an intersection between cell-specific DNA methylation dynamics and addiction susceptibility. Drugs of abuse have been shown to promote the formation of neuronal ensembles in rodent brain (e.g., NAc, amygdala, and cortex), and their synchronous activation facilitates stimuli-specific behavioral plasticity and memory retrieval (Cruz et al., 2013; de Guglielmo et al., 2016; Nawarawong and Olsen, 2020; Sun et al., 2020). A recent study also found that DNA methylation affected neuronal ensemble formation. Dnmt3a over-expression in mouse hippocampal ensembles enhanced the retrieval of memories in fear-conditioned mice, and its over-expression induced hypermethylation at synaptic plasticity genes in recently-activated cultured hippocampal neurons (Karaca et al., 2020). The contribution of DNA methylation to neural plasticity in neuronal ensembles will likely give rise to characterize addiction. This may represent a functional connection between cell type-specific DNA modifications and addiction susceptibility.

There may be significant differences in DNA methylation patterns in different brain regions and cell types in the SUDs. Previous studies have focused primarily on brain region-specific explorations. However, brain regions contain multiple cell types, and it remains an open question as to which cell types DNA methylation occurs in and how it occurs. Additionally, DNA methylation assays are becoming more accurate and affordable and can be used on various tissue types and species. Due to the rapid development of technology in recent years, such as Single-Cell Sequencing (SCS), Mass Spectrometry Flow Cytometry (CyTOF), Near Infrared Spectroscopy (fNIRS), Gas Chromatography-Mass Spectrometry (GC-MS), High-Performance Liquid Chromatography (HPLC) and MethyLasso (Balaramane et al., 2024), it is expected that we can accurately identify which specific cell within the brain region has undergone DNA methylation changes, thus further revealing the mechanism of DNA methylation in the SUDs.

The central nervous system has a high metabolic rate because neurons need an enormous amount of energy for maintenance of ionic gradients across the cell membrane and for neurotransmission and synaptic plasticity (Kann and Kovács, 2007). Synaptic transmission re quires mitochondrial ATP generation that greatly depends on mitochondrial function and oxygen supply. It is suggested that mtDNA changes and mitochondrial dysfunction may affect neurotransmission (Streck et al., 1999) and are actively involved in the process of drug ad diction (Feng et al., 2013). Feng et al. (2013) has demonstrated that chronic morphine exposure decreased mtDNA copy number in the rat hippocampus. This study also found that heroin addicts had a lower level of mtDNA copy number in the peripheral blood. The rat pheochromocytoma cells, after morphine treatment, showed decreased mtDNA copy number and elevated levels of ROS as well as increased mitochondrial mass (which may reflect mitochondrial dysfunction). The authors speculate that ROS would trigger autophagy to eliminate dysfunctional mitochondria and lead to a reduction of mtDNA copy number (Feng et al., 2013). Another study has reported that even a high dose of morphine is well tolerated by brain mitochondria probably due to the antioxidant effect of this drug (Cunha-Oliveira et al., 2013). The discrepancy in these findings suggests that the effects of drugs on mitochondrial function may be modulated by multiple factors. Further, a short report from the Iranian Prenatal METH Exposure Effects Cohort revealed another level of impact: DNA methylation of genes related to mitochondrial function was also altered, suggesting the possibility that METH may have a long-range effect on gene expression early in embryonic development (Haghighatfard et al., 2022). DNA methylation is dynamic, which changes with environment, age, and intake of potent abused drugs; hence, a keen eye is needed to keep up with the upcoming scientific discoveries in the field. In recent years, a growing body of scientific evidence has emphasized the importance of research on the balance between DNA methylation and demethylation. This raises a thought-provoking question: is it possible that the combined application of methylation inhibitors and demethylation agonist may provide a more effective intervention for the SUDs? In addition, current research has focused on the function of DNMT and TET family enzymes, and we look forward to more in-depth studies of these and other related enzymes in the future to more fully understand the role of DNA methylation and demethylation in the SUDs.

Intergenerational genetic effects have also been a hot topic in SUD research in recent years. Parental or maternal alcohol and drug dependence can affect the overall health of offsprings and predispose them to various diseases such as cancer or drug addiction, which can be utilized in public health awareness or preventive initiatives. The consistency in the pattern of association between change in DNA methylation and drug dependence calls for the need to understand these mechanisms with a deeper insight and extensively to develop it as a better biomarker for drug methylation effects in future epidemiology and forensic studies, as well. However, in terms of the interaction between DNA methylation mechanisms and genetic factors, the current level of research has not yet been able to fully resolve this complex issue due to the long research span and difficulty in tracking. For example, although evidence have demonstrated that drug addiction indeed changes the DNA methylation within germ cells in F0 and F1 animals (Youngson and Whitelaw, 2008; Vassoler et al., 2014; Le et al., 2017), the specific ways these changes impact brain function and behaviors related to addiction remain unclear. To uncover these mechanism, we need to further track the overall view of DNA methylation in entire developmental process from germ cells to adult animals, which is beyond the scope of this review. It remains a great challenge to effectively detect genes that continue to differentiate during the genetic process. Addiction calls for the need for multidisciplinary contribution. We expect that more effective detection methods and technologies will be developed in the future to probe more deeply into the role of DNA methylation mechanisms in the inheritance of the SUDs and to provide new breakthroughs for scientific research in this field.

Note that although studies in animal models have offered valuable insights into the biological basis of addiction, there is still a long way to go from animal research to clinical application. Firstly, it is essential to perform more human research, including a detailed examination of the brains of deceased individuals who struggled with addiction, to confirm the addiction-related changes at the molecular, cellular, and brain-region levels observed in animal models. Secondly, if the DNA methylation mechanisms were further confirmed in addicts, developing precise treatment strategies and reducing side effects of treatment base on DNA methylation are still a primary challenge. In particular, minimizing off-target effects. This necessitates thorough *in vitro* and *in vivo* screening during the drug development phase, the use of advanced bioinformatics tools to predict drug binding properties and ranges of action, as well as rigorous clinical trials to monitor and assess side effects. Thirdly, found the peripheral DNA methylation markers and confirm its relevance to addictive behavioral phenotypes. A study have found significant overlap of hypomethylated gene between the T cells in humans and NAc in rats (Massart et al., 2015), supporting that peripheral T cells are highly informative on DNA methylation that occur in the brain and are associated with SUD.

The potential off-target effects can complicate the results when trying to reverse hypermethylation caused by drugs. However, targeted DNA cleavage techniques, such as CRISPR-Cas9 system, transcription activator-like effector nucleases (TALENs) and zinc-finger nucleases (ZFNs), have fused with epigenetic writer or eraser proteins and may bring hope for solving this problem. Take CRISPR-Cas9 system for example, the advanced engineered Protospacer Adjacent Motif (PAMs) has greatly expanded the repertoire of prospective CRISPR/Cas target sequences (Doench et al., 2016). Furthermore, a recent study has found unique nanocapsule-based CRISPR-Cas9 delivery system, which is non-invasive, facilitates BBB penetration and safe for glioblastoma gene therapy (Zou et al., 2022). This advancements in CRISPR-Cas9 delivery at brain cells, are hopefully expand to epigenetic therapy, including genes targeted by DNMTs. The development of agents with multiple high affinity epigenetic targets, including epigenetic enzyme (Lera and Ganesan, 2020), also reduces adverse drug reactions (Tomaselli et al., 2020). In the light of such developments, advancement in targeted epigenetic remodeling or DNA cleavage techniques with fused or engineered epigenetic enzymes, small-molecule epigenetic modulators, nanoparticle-based, viral-mediated, peptide-based, and receptor-based drug delivery systems with ability to cross blood-brain barrier is expected for addiction treatment.

## References

[B1] Aapola U. Kawasaki K. Scott H. Ollila J. Vihinen M. Heino M. (2000). Isolation and initial characterization of a novel zinc finger gene, DNMT3L, on 21q22.3, related to the cytosine-5-methyltransferase 3 gene family. *Genomics* 65 293–298. 10.1006/geno.2000.6168 10857753

[B2] Agulló L. Escorial M. Orutño S. Muriel J. Sandoval J. Margarit C. (2024). Epigenetic and sex differences in opioid use disorder in chronic pain: A real-world study linked with OPRM1 DNA methylation. *Addict. Biol.* 29:e13422. 10.1111/adb.13422 38949208 PMC11215788

[B3] Ajonijebu D. Abboussi O. Mabandla M. Daniels W. (2019). Cocaine-induced inheritable epigenetic marks may be altered by changing early postnatal fostering. *Neuroreport* 30 1157–1165. 10.1097/WNR.0000000000001332 31568187

[B4] Ajonijebu D. C. Abboussi O. Mabandla M. V. Daniels W. M. U. (2018). Differential epigenetic changes in the hippocampus and prefrontal cortex of female mice that had free access to cocaine. *Metab. Brain Dis.* 33 411–420. 10.1007/s11011-017-0116-z 28963688

[B5] Alaghband Y. Bredy T. Wood M. (2016). The role of active DNA demethylation and Tet enzyme function in memory formation and cocaine action. *Neurosci. Lett.* 625 40–46. 10.1016/j.neulet.2016.01.023 26806038 PMC4903882

[B6] Albertson D. Pruetz B. Schmidt C. Kuhn D. Kapatos G. Bannon M. (2004). Gene expression profile of the nucleus accumbens of human cocaine abusers: Evidence for dysregulation of myelin. *J. Neurochem.* 88 1211–1219. 10.1046/j.1471-4159.2003.02247.x 15009677 PMC2215309

[B7] Anier K. Malinovskaja K. Aonurm-Helm A. Zharkovsky A. Kalda A. D. N. A. (2010). methylation regulates cocaine-induced behavioral sensitization in mice. *Neuropsychopharmacology* 35 2450–2461. 10.1038/npp.2010.128 20720536 PMC3055323

[B8] Anier K. Urb M. Kipper K. Herodes K. Timmusk T. Zharkovsky A. (2018). Cocaine-induced epigenetic DNA modification in mouse addiction-specific and non-specific tissues. *Neuropharmacology* 139 13–25. 10.1016/j.neuropharm.2018.06.036 29964092

[B9] Anier K. Zharkovsky A. Kalda A. S. - (2013). adenosylmethionine modifies cocaine-induced DNA methylation and increases locomotor sensitization in mice. *Int. J. Neuropsychopharmacol.* 16 2053–2066. 10.1017/S1461145713000394 23684129

[B10] Auber A. Tedesco V. Jones C. Monfils M. Chiamulera C. (2013). Post-retrieval extinction as reconsolidation interference: Methodological issues or boundary conditions? *Psychopharmacology (Berl)* 226 631–647. 10.1007/s00213-013-3004-1 23404065 PMC3682675

[B11] Baker-Andresen D. Zhao Q. Li X. Jupp B. Chesworth R. Lawrence A. (2015). Persistent variations in neuronal DNA methylation following cocaine self-administration and protracted abstinence in mice. *Neuroepigenetics* 4 1–11. 10.1016/j.nepig.2015.10.001 27213137 PMC4874530

[B12] Balaramane D. Spill Y. Weber M. Bardet A. (2024). MethyLasso: A segmentation approach to analyze DNA methylation patterns and identify differentially methylated regions from whole-genome datasets. *Nucleic Acids Res.* 52:e98. 10.1093/nar/gkae880 39420630 PMC11602171

[B13] Berger S. Kouzarides T. Shiekhattar R. Shilatifard A. (2009). An operational definition of epigenetics. *Genes Dev.* 23 781–783. 10.1101/gad.1787609 19339683 PMC3959995

[B14] Berke J. Hyman S. (2000). Addiction, dopamine, and the molecular mechanisms of memory. *Neuron* 25 515–532. 10.1016/s0896-6273(00)81056-9 10774721

[B15] Biagioni F. Ferese R. Limanaqi F. Madonna M. Lenzi P. Gambardella S. (2019). Methamphetamine persistently increases alpha-synuclein and suppresses gene promoter methylation within striatal neurons. *Brain Res.* 1719 157–175. 10.1016/j.brainres.2019.05.035 31150652

[B16] Bodetto S. Carouge D. Fonteneau M. Dietrich J. Zwiller J. Anglard P. (2013). Cocaine represses protein phosphatase-1Cβ through DNA methylation and Methyl-CpG Binding Protein-2 recruitment in adult rat brain. *Neuropharmacology* 73 31–40. 10.1016/j.neuropharm.2013.05.005 23688924

[B17] Boschen K. Keller S. Roth T. Klintsova A. (2018). Epigenetic mechanisms in alcohol- and adversity-induced developmental origins of neurobehavioral functioning. *Neurotoxicol. Teratol.* 66 63–79. 10.1016/j.ntt.2017.12.009 29305195 PMC5856624

[B18] Bourtchouladze R. Patterson S. Kelly M. Kreibich A. Kandel E. Abel T. (2006). Chronically increased Gsalpha signaling disrupts associative and spatial learning. *Learn. Mem.* 13 745–752. 10.1101/lm.354106 17142304 PMC1783628

[B19] Cadet J. Brannock C. Krasnova I. Jayanthi S. Ladenheim B. McCoy M. (2017). Genome-wide DNA hydroxymethylation identifies potassium channels in the nucleus accumbens as discriminators of methamphetamine addiction and abstinence. *Mol. Psychiatry* 22 1196–1204. 10.1038/mp.2016.48 27046646 PMC7405865

[B20] Cadet J. L. Patel R. Jayanthi S. (2019). Compulsive methamphetamine taking and abstinence in the presence of adverse consequences: Epigenetic and transcriptional consequences in the rat brain. *Pharmacol. Biochem. Behav.* 179 98–108. 10.1016/j.pbb.2019.02.009 30797763

[B21] Camerota M. Davis J. Dansereau L. Oliveira E. Padbury J. Lester B. (2022). Effects of pharmacologic treatment for neonatal abstinence syndrome on DNA methylation and neurobehavior: A prospective cohort study. *J. Pediatr.* 243 21–26. 10.1016/j.jpeds.2021.12.057 34971656 PMC8960328

[B22] Cannella N. Oliveira A. Hemstedt T. Lissek T. Buechler E. Bading H. (2018). Dnmt3a2 in the nucleus accumbens shell is required for reinstatement of cocaine seeking. *J. Neurosci.* 38 7516–7528. 10.1523/JNEUROSCI.0600-18.2018 30030395 PMC6596133

[B23] Carouge D. Host L. Aunis D. Zwiller J. Anglard P. (2010). CDKL5 is a brain MeCP2 target gene regulated by DNA methylation. *Neurobiol. Dis.* 38 414–424. 10.1016/j.nbd.2010.02.014 20211261

[B24] Carrico A. Cherenack E. Flentje A. Moskowitz J. Asam K. Ghanooni D. (2024). A positive affect intervention alters leukocyte DNA methylation in sexual minority men with HIV who use methamphetamine. *Brain Behav. Immun.* 120 151–158. 10.1016/j.bbi.2024.05.025 38777283 PMC11269022

[B25] Chao M. Fragou D. Zanos P. Hu C. Bailey A. Kouidou S. (2014). Epigenetically modified nucleotides in chronic heroin and cocaine treated mice. *Toxicol. Lett.* 229 451–457. 10.1016/j.toxlet.2014.07.023 25064621

[B26] Chao Y. Xie F. Li X. Guo R. Yang N. Zhang C. (2016). Demethylation regulation of BDNF gene expression in dorsal root ganglion neurons is implicated in opioid-induced pain hypersensitivity in rats. *Neurochem. Int.* 97 91–98. 10.1016/j.neuint.2016.03.007 26970395

[B27] Chen Z. Wang Y. Chen R. Geng F. Gan C. Wang W. (2021). Ube2b-dependent degradation of DNMT3a relieves a transcriptional brake on opiate-induced synaptic and behavioral plasticity. *Mol. Psychiatry* 26 1162–1177. 10.1038/s41380-019-0533-y 31576007

[B28] Chorbov V. Todorov A. Lynskey M. Cicero T. (2011). Elevated levels of DNA methylation at the OPRM1 promoter in blood and sperm from male opioid addicts. *J. Opioid. Manag.* 7 258–264. 10.5055/jom.2011.0067 21957825 PMC4142589

[B29] Cruz F. Koya E. Guez-Barber D. Bossert J. Lupica C. Shaham Y. (2013). New technologies for examining the role of neuronal ensembles in drug addiction and fear. *Nat. Rev. Neurosci.* 14 743–754. 10.1038/nrn3597 24088811 PMC4530016

[B30] Cunha-Oliveira T. Silva L. Silva A. Moreno A. Oliveira C. Santos M. (2013). Mitochondrial complex I dysfunction induced by cocaine and cocaine plus morphine in brain and liver mitochondria. *Toxicol. Lett.* 219 298–306. 10.1016/j.toxlet.2013.03.025 23542814

[B31] Day J. Sweatt J. (2011). Cognitive neuroepigenetics: A role for epigenetic mechanisms in learning and memory. *Neurobiol. Learn. Mem.* 96 2–12. 10.1016/j.nlm.2010.12.008 21195202 PMC3111867

[B32] de Guglielmo G. Crawford E. Kim S. Vendruscolo L. Hope B. Brennan M. (2016). Recruitment of a neuronal ensemble in the central nucleus of the amygdala is required for alcohol dependence. *J. Neurosci.* 36 9446–9453. 10.1523/JNEUROSCI.1395-16.2016 27605618 PMC5013191

[B33] Doehring A. Oertel B. Sittl R. Lötsch J. (2013). Chronic opioid use is associated with increased DNA methylation correlating with increased clinical pain. *Pain* 154 15–23. 10.1016/j.pain.2012.06.011 23273101

[B34] Doench J. Fusi N. Sullender M. Hegde M. Vaimberg E. Donovan K. (2016). Optimized sgRNA design to maximize activity and minimize off-target effects of CRISPR-Cas9. *Nat. Biotechnol.* 34 184–191. 10.1038/nbt.3437 26780180 PMC4744125

[B35] Doke M. Jeganathan V. McLaughlin J. Samikkannu T. (2021). HIV-1 Tat and cocaine impact mitochondrial epigenetics: Effects on DNA methylation. *Epigenetics* 16 980–999. 10.1080/15592294.2020.1834919 33100130 PMC8451453

[B36] Dong N. Zhu J. Wang R. Wang S. Chen Y. Wang C. (2022). Maternal methamphetamine exposure influences behavioral sensitization and nucleus accumbens DNA methylation in subsequent generation. *Front. Pharmacol.* 13:940798. 10.3389/fphar.2022.940798 35928279 PMC9343784

[B37] Dumitriu D. Laplant Q. Grossman Y. Dias C. Janssen W. Russo S. (2012). Subregional, dendritic compartment, and spine subtype specificity in cocaine regulation of dendritic spines in the nucleus accumbens. *J. Neurosci.* 32 6957–6966. 10.1523/JNEUROSCI.5718-11.2012 22593064 PMC3360066

[B38] Ebrahimi G. Asadikaram G. Akbari H. Nematollahi M. Abolhassani M. Shahabinejad G. (2018). Elevated levels of DNA methylation at the OPRM1 promoter region in men with opioid use disorder. *Am. J. Drug Alcohol. Abuse* 44 193–199. 10.1080/00952990.2016.1275659 28121474

[B39] Fan B. Hao B. Dai Y. Xue L. Shi Y. Liu L. (2022). Deficiency of Tet3 in nucleus accumbens enhances fear generalization and anxiety-like behaviors in mice. *Brain Pathol.* 32:e13080. 10.1111/bpa.13080 35612904 PMC9616092

[B40] Fan X. Shi G. Zhao P. (2019). Methylation in Syn and Psd95 genes underlie the inhibitory effect of oxytocin on oxycodone-induced conditioned place preference. *Eur. Neuropsychopharmacol.* 29 1464–1475. 10.1016/j.euroneuro.2019.10.010 31735530

[B41] Fan X. Shi G. He X. Li X. Wan Y. Jian L. (2021). Oxytocin prevents cue-induced reinstatement of oxycodone seeking: Involvement of DNA methylation in the hippocampus. *Addict. Biol.* 26:e13025. 10.1111/adb.13025 33609013

[B42] Fan X. Yang J. Dong Y. Hou Y. Liu S. Wu C. (2020). Oxytocin inhibits methamphetamine-associated learning and memory alterations by regulating DNA methylation at the Synaptophysin promoter. *Addict. Biol.* 25:e12697. 10.1111/adb.12697 30585381

[B43] Farris S. Mayfield R. (2021). Epigenetic and non-coding regulation of alcohol abuse and addiction. *Int. Rev. Neurobiol.* 156 63–86. 10.1016/bs.irn.2020.08.006 33461665 PMC8939284

[B44] Feng J. Shao N. Szulwach K. Vialou V. Huynh J. Zhong C. (2015). Role of Tet1 and 5-hydroxymethylcytosine in cocaine action. *Nat. Neurosci.* 18 536–544. 10.1038/nn.3976 25774451 PMC4617315

[B45] Feng Y. Jia Y. Su L. Wang D. Lv L. Xu L. (2013). Decreased mitochondrial DNA copy number in the hippocampus and peripheral blood during opiate addiction is mediated by autophagy and can be salvaged by melatonin. *Autophagy* 9 1395–1406. 10.4161/auto.25468 23800874

[B46] Ferrer-Alcón M. La Harpe R. García-Sevilla J. (2004). Decreased immunodensities of micro-opioid receptors, receptor kinases GRK 2/6 and beta-arrestin-2 in postmortem brains of opiate addicts. *Brain Res. Mol. Brain Res.* 121 114–122. 10.1016/j.molbrainres.2003.11.009 14969742

[B47] First M. (2013). Diagnostic and statistical manual of mental disorders, 5th edition, and clinical utility. *J. Nerv. Ment. Dis.* 201 727–729. 10.1097/NMD.0b013e3182a2168a 23995026

[B48] Fonteneau M. Filliol D. Anglard P. Befort K. Romieu P. Zwiller J. (2017). Inhibition of DNA methyltransferases regulates cocaine self-administration by rats: A genome-wide DNA methylation study. *Genes Brain Behav.* 16 313–327. 10.1111/gbb.12354 27762100

[B49] Fragou D. Zanos P. Kouidou S. Njau S. Kitchen I. Bailey A. (2013). Effect of chronic heroin and cocaine administration on global DNA methylation in brain and liver. *Toxicol. Lett.* 218 260–265. 10.1016/j.toxlet.2013.01.022 23454526

[B50] Fuchs R. Evans K. Ledford C. Parker M. Case J. Mehta R. (2005). The role of the dorsomedial prefrontal cortex, basolateral amygdala, and dorsal hippocampus in contextual reinstatement of cocaine seeking in rats. *Neuropsychopharmacology* 30 296–309. 10.1038/sj.npp.1300579 15483559

[B51] García-Sevilla J. Ventayol P. Busquets X. La Harpe R. Walzer C. Guimón J. (1997). Regulation of immunolabelled mu-opioid receptors and protein kinase C-alpha and zeta isoforms in the frontal cortex of human opiate addicts. *Neurosci. Lett.* 226 29–32. 10.1016/s0304-3940(97)00227-9 9153634

[B52] Goldberg A. Allis C. Bernstein E. (2007). Epigenetics: A landscape takes shape. *Cell* 128 635–638. 10.1016/j.cell.2007.02.006 17320500

[B53] Gonçalves J. Martins T. Ferreira R. Milhazes N. Borges F. Ribeiro C. (2008). Methamphetamine-induced early increase of IL-6 and TNF-alpha mRNA expression in the mouse brain. *Ann. N. Y. Acad. Sci.* 1139 103–111. 10.1196/annals.1432.043 18991854

[B54] González B. Jayanthi S. Gomez N. Torres O. Sosa M. Bernardi A. (2018). Repeated methamphetamine and modafinil induce differential cognitive effects and specific histone acetylation and DNA methylation profiles in the mouse medial prefrontal cortex. *Prog. Neuropsychopharmacol. Biol. Psychiatry* 82 1–11. 10.1016/j.pnpbp.2017.12.009 29247759 PMC6983674

[B55] Greenberg M. Bourc’his D. (2019). The diverse roles of DNA methylation in mammalian development and disease. *Nat. Rev. Mol. Cell. Biol.* 20 590–607. 10.1038/s41580-019-0159-6 31399642

[B56] Guibert S. Forné T. Weber M. (2012). Global profiling of DNA methylation erasure in mouse primordial germ cells. *Genome Res.* 22 633–641. 10.1101/gr.130997.111 22357612 PMC3317146

[B57] Guo J. Su Y. Zhong C. Ming G. Song H. (2011). Hydroxylation of 5-methylcytosine by TET1 promotes active DNA demethylation in the adult brain. *Cell* 145 423–434. 10.1016/j.cell.2011.03.022 21496894 PMC3088758

[B58] Guo M. Liao K. Periyasamy P. Yang L. Cai Y. Callen S. (2015). Cocaine-mediated microglial activation involves the ER stress-autophagy axis. *Autophagy* 11 995–1009. 10.1080/15548627.2015.1052205 26043790 PMC4590604

[B59] Guo M. Periyasamy P. Liao K. Kook Y. Niu F. Callen S. (2016). Cocaine-mediated downregulation of microglial miR-124 expression involves promoter DNA methylation. *Epigenetics* 11 819–830. 10.1080/15592294.2016.1232233 27786595 PMC5221603

[B60] Hackett J. Sengupta R. Zylicz J. Murakami K. Lee C. Down T. (2013). Germline DNA demethylation dynamics and imprint erasure through 5-hydroxymethylcytosine. *Science* 339 448–452. 10.1126/science.1229277 23223451 PMC3847602

[B61] Haddar M. Uno K. Azuma K. Muramatsu S. Nitta A. (2020). Inhibitory effects of Shati/Nat8l overexpression in the medial prefrontal cortex on methamphetamine-induced conditioned place preference in mice. *Addict. Biol.* 25:e12749. 10.1111/adb.12749 30950164 PMC7187255

[B62] Haghighatfard A. Salehi M. Saberi S. Hashemi M. (2022). Short report of an Iranian prenatal methamphetamine exposure effect cohort showed DNA methylation alteration of mitochondria function associated genes. *Res. Dev. Disabil.* 129:104320. 10.1016/j.ridd.2022.104320 35930865

[B63] Han J. Li Y. Wang D. Wei C. Yang X. Sui N. (2010). Effect of 5-aza-2-deoxycytidine microinjecting into hippocampus and prelimbic cortex on acquisition and retrieval of cocaine-induced place preference in C57BL/6 mice. *Eur. J. Pharmacol.* 642 93–98. 10.1016/j.ejphar.2010.05.050 20550947

[B64] Hao L. Luo T. Dong H. Tang A. Hao W. (2017). CHN2 promoter methylation change may be associated with methamphetamine dependence. *Shanghai Arch. Psychiatry* 29 357–364. 10.11919/j.issn.1002-0829.217100 29719347 PMC5925587

[B65] Hata K. Okano M. Lei H. Li E. (2002). Dnmt3L cooperates with the Dnmt3 family of de novo DNA methyltransferases to establish maternal imprints in mice. *Development* 129 1983–1993. 10.1242/dev.129.8.1983 11934864

[B66] He F. Lidow I. Lidow M. (2006). Consequences of paternal cocaine exposure in mice. *Neurotoxicol. Teratol.* 28 198–209. 10.1016/j.ntt.2005.12.003 16458479

[B67] Heath A. Bucholz K. Madden P. Dinwiddie S. Slutske W. Bierut L. (1997). Genetic and environmental contributions to alcohol dependence risk in a national twin sample: Consistency of findings in women and men. *Psychol. Med.* 27 1381–1396. 10.1017/s0033291797005643 9403910

[B68] Héberlé É Bardet A. F. (2019). Sensitivity of transcription factors to DNA methylation. *Essays Biochem.* 63 727–741. 10.1042/EBC20190033 31755929 PMC6923324

[B69] Heiman M. Schaefer A. Gong S. Peterson J. Day M. Ramsey K. (2008). A translational profiling approach for the molecular characterization of CNS cell types. *Cell* 135 738–748. 10.1016/j.cell.2008.10.028 19013281 PMC2696821

[B70] Host L. Dietrich J. Carouge D. Aunis D. Zwiller J. (2011). Cocaine self-administration alters the expression of chromatin-remodelling proteins; modulation by histone deacetylase inhibition. *J. Psychopharmacol.* 25 222–229. 10.1177/0269881109348173 19939859

[B71] Iamjan S. Thanoi S. Watiktinkorn P. Fachim H. Dalton C. Nudmamud-Thanoi S. (2021). Changes of BDNF exon IV DNA methylation are associated with methamphetamine dependence. *Epigenomics* 13 953–965. 10.2217/epi-2020-0463 34008409

[B72] Iamjan S. Veerasakul S. Reynolds G. Thanoi S. Nudmamud-Thanoi S. (2024). Regional-specific changes in rat brain BDNF in a model of methamphetamine abuse. *Neurosci. Lett.* 836:137880. 10.1016/j.neulet.2024.137880 38885757

[B73] Im H. Hollander J. Bali P. Kenny P. (2010). MeCP2 controls BDNF expression and cocaine intake through homeostatic interactions with microRNA-212. *Nat. Neurosci.* 13 1120–1127. 10.1038/nn.2615 20711185 PMC2928848

[B74] Ito S. Shen L. Dai Q. Wu S. Collins L. Swenberg J. (2011). Tet proteins can convert 5-methylcytosine to 5-formylcytosine and 5-carboxylcytosine. *Science* 333 1300–1303. 10.1126/science.1210597 21778364 PMC3495246

[B75] Itzhak Y. Ergui I. Young J. (2015). Long-term parental methamphetamine exposure of mice influences behavior and hippocampal DNA methylation of the offspring. *Mol. Psychiatry* 20 232–239. 10.1038/mp.2014.7 24535458

[B76] Jayanthi S. Gonzalez B. McCoy M. Ladenheim B. Bisagno V. Cadet J. (2018). Methamphetamine Induces TET1- and TET3-dependent DNA Hydroxymethylation of Crh and Avp genes in the rat nucleus accumbens. *Mol. Neurobiol.* 55 5154–5166. 10.1007/s12035-017-0750-9 28842817 PMC5948251

[B77] Jayanthi S. McCoy M. Chen B. Britt J. Kourrich S. Yau H. (2014). Methamphetamine downregulates striatal glutamate receptors via diverse epigenetic mechanisms. *Biol. Psychiatry* 76 47–56. 10.1016/j.biopsych.2013.09.034 24239129 PMC3989474

[B78] Jayanthi S. Torres O. Ladenheim B. Cadet J. L. A. (2020). Single prior injection of methamphetamine enhances methamphetamine self-administration (SA) and Blocks SA-induced changes in DNA methylation and mrna expression of potassium channels in the rat nucleus accumbens. *Mol. Neurobiol.* 57 1459–1472. 10.1007/s12035-019-01830-3 31758400 PMC7060962

[B79] Jiang F. Zheng W. Wu C. Li Y. Shen F. Liang J. (2021). Double dissociation of inhibitory effects between the hippocampal TET1 and TET3 in the acquisition of morphine self-administration in rats. *Addict. Biol.* 26:e12875. 10.1111/adb.12875 32031744

[B80] Joffe M. Grueter C. Grueter B. (2014). Biological substrates of addiction. *Wiley Interdiscip. Rev. Cogn. Sci.* 5 151–171. 10.1002/wcs.1273 24999377 PMC4078878

[B81] Kaas G. Zhong C. Eason D. Ross D. Vachhani R. Ming G. (2013). TET1 controls CNS 5-methylcytosine hydroxylation, active DNA demethylation, gene transcription, and memory formation. *Neuron* 79 1086–1093. 10.1016/j.neuron.2013.08.032 24050399 PMC3816951

[B82] Kann O. Kovács R. (2007). Mitochondria and neuronal activity. *Am. J. Physiol. Cell. Physiol.* 292 C641–C657. 10.1152/ajpcell.00222.2006 17092996

[B83] Karaca G. K. Kupke J. Brito D. Zeuch B. Thome C. Weichenhan D. (2020). Neuronal ensemble-specific DNA methylation strengthens engram stability. *Nat. Commun.* 11:639. 10.1038/s41467-020-14498-4 32005851 PMC6994722

[B84] Kauer J. Malenka R. (2007). Synaptic plasticity and addiction. *Nat. Rev. Neurosci.* 8 844–858. 10.1038/nrn2234 17948030

[B85] Kenny P. (2014). Epigenetics, microRNA, and addiction. *Dialogues Clin. Neurosci.* 16 335–344. 10.31887/DCNS.2014.16.3/pkenny 25364284 PMC4214176

[B86] Kim M. Costello J. (2017). DNA methylation: An epigenetic mark of cellular memory. *Exp. Mol. Med.* 49:e322. 10.1038/emm.2017.10 28450738 PMC6130213

[B87] Kreek M. Levran O. Reed B. Schlussman S. Zhou Y. Butelman E. (2012). Opiate addiction and cocaine addiction: Underlying molecular neurobiology and genetics. *J. Clin. Invest.* 122 3387–3393. 10.1172/JCI60390 23023708 PMC3534165

[B88] Kristiansen L. Bannon M. Meador-Woodruff J. (2009). Expression of transcripts for myelin related genes in postmortem brain from cocaine abusers. *Neurochem. Res.* 34 46–54. 10.1007/s11064-008-9655-3 18357522 PMC2615829

[B89] Kulis M. Esteller M. (2010). DNA methylation and cancer. *Adv. Genet.* 70 27–56. 10.1016/B978-0-12-380866-0.60002-2 20920744

[B90] Lambert B. Bauer C. (2012). Developmental and behavioral consequences of prenatal cocaine exposure: A review. *J. Perinatol.* 32 819–828. 10.1038/jp.2012.90 22791278 PMC4143247

[B91] LaPlant Q. Vialou V. Covington H. Dumitriu D. Feng J. Warren B. (2010). Dnmt3a regulates emotional behavior and spine plasticity in the nucleus accumbens. *Nat. Neurosci.* 13 1137–1143. 10.1038/nn.2619 20729844 PMC2928863

[B92] Le Q. Yan B. Yu X. Li Y. Song H. Zhu H. (2017). Drug-seeking motivation level in male rats determines offspring susceptibility or resistance to cocaine-seeking behaviour. *Nat. Commun.* 8:15527. 10.1038/ncomms15527 28556835 PMC5459992

[B93] Lera A. Ganesan A. (2020). Two-hit wonders: The expanding universe of multitargeting epigenetic agents. *Curr. Opin. Chem. Biol.* 57 135–154. 10.1016/j.cbpa.2020.05.009 32784072

[B94] Li S. Papale L. Kintner D. Sabat G. Barrett-Wilt G. Cengiz P. (2015). Hippocampal increase of 5-hmC in the glucocorticoid receptor gene following acute stress. *Behav. Brain Res.* 286 236–240. 10.1016/j.bbr.2015.03.002 25746451 PMC4398338

[B95] Li X. Wei W. Ratnu V. Bredy T. (2013). On the potential role of active DNA demethylation in establishing epigenetic states associated with neural plasticity and memory. *Neurobiol. Learn. Mem.* 105 125–132. 10.1016/j.nlm.2013.06.009 23806749

[B96] Li Y. Lu Y. Xie Q. Zeng X. Zhang R. Dang W. (2022). Methylation and expression quantitative trait locus rs6296 in the HTR1B gene is associated with susceptibility to opioid use disorder. *Psychopharmacology (Berl)* 239 2515–2523. 10.1007/s00213-022-06141-5 35438303

[B97] Li Z. Liu D. Wang G. Zheng Y. Chen L. Cheng Z. (2024). METH exposure alters sperm DNA methylation in F0 mice and mPFC transcriptome in male F1 mice. *Psychopharmacology (Berl)* 241 897–911. 10.1007/s00213-023-06516-2 38092953

[B98] Liao K. Guo M. Niu F. Yang L. Callen S. Buch S. (2016). Cocaine-mediated induction of microglial activation involves the ER stress-TLR2 axis. *J. Neuroinflammation* 13:33. 10.1186/s12974-016-0501-2 26860188 PMC4748483

[B99] Liu A. Dai Y. Mendez E. Hu R. Fries G. Najera K. (2021). Genome-Wide correlation of dna methylation and gene expression in postmortem brain tissues of opioid use disorder patients. *Int. J. Neuropsychopharmacol.* 24 879–891. 10.1093/ijnp/pyab043 34214162 PMC8598308

[B100] Liu C. Sun X. Wang Z. Le Q. Liu P. Jiang C. (2018). Retrieval-induced upregulation of Tet3 in pyramidal neurons of the dorsal hippocampus mediates cocaine-associated memory reconsolidation. *Int. J. Neuropsychopharmacol.* 21 255–266. 10.1093/ijnp/pyx099 29106571 PMC5838812

[B101] Liu P. Liang J. Jiang F. Cai W. Shen F. Liang J. (2022). Gnas promoter hypermethylation in the basolateral amygdala regulates reconsolidation of morphine reward memory in rats. *Genes (Basel)* 13:553. 10.3390/genes13030553 35328106 PMC8950747

[B102] Liu P. Zhang J. Li M. Sui N. (2016). Distinctive roles of 5-aza-2’-deoxycytidine in anterior agranular insular and basolateral amygdala in reconsolidation of aversive memory associated with morphine in rats. *Front. Behav. Neurosci.* 10:50. 10.3389/fnbeh.2016.00050 27014010 PMC4791382

[B103] Lukowiak K. Heckler B. Bennett T. Schriner E. Wyrick K. Jewett C. (2014). Enhanced memory persistence is blocked by a DNA methyltransferase inhibitor in the snail Lymnaea stagnalis. *J. Exp. Biol.* 217 2920–2929. 10.1242/jeb.106765 24902747

[B104] MacDonald J. Jackson M. Beazely M. A. (2007). G protein-coupled receptors control NMDARs and metaplasticity in the hippocampus. *Biochim. Biophys. Acta* 1768 941–951. 10.1016/j.bbamem.2006.12.006 17261268

[B105] Mahna D. Puri S. Sharma S. D. N. (2018). A methylation signatures: biomarkers of drug and alcohol abuse. *Mutat. Res. Rev. Mutat. Res.* 777 19–28. 10.1016/j.mrrev.2018.06.002 30115428

[B106] Massart R. Barnea R. Dikshtein Y. Suderman M. Meir O. Hallett M. (2015). Role of DNA methylation in the nucleus accumbens in incubation of cocaine craving. *J. Neurosci.* 35 8042–8058. 10.1523/JNEUROSCI.3053-14.2015 26019323 PMC6605346

[B107] McCoy M. Jayanthi S. Cadet J. (2021). Potassium channels and their potential roles in substance use disorders. *Int. J. Mol. Sci.* 22:1249. 10.3390/ijms22031249 33513859 PMC7865894

[B108] McDonough J. Singhal N. Getsy P. Knies K. Knauss Z. Mueller D. (2024). The epigenetic signatures of opioid addiction and physical dependence are prevented by D-cysteine ethyl ester and betaine. *Front. Pharmacol.* 15:1416701. 10.3389/fphar.2024.1416701 39281282 PMC11392886

[B109] Melo C. O. D. Cidália Vieira T. Duarte Gigonzac M. Soares Fortes J. Moreira Duarte S. da Cruz A. (2020). Evaluation of polymorphisms in repair and detoxification genes in alcohol drinkers and non-drinkers using capillary electrophoresis. *Electrophoresis* 41 254–258. 10.1002/elps.201900193 31886888

[B110] Miller C. Sweatt J. (2007). Covalent modification of DNA regulates memory formation. *Neuron* 53 857–869. 10.1016/j.neuron.2007.02.022 17359920

[B111] Miller C. Campbell S. Sweatt J. D. (2008). DNA methylation and histone acetylation work in concert to regulate memory formation and synaptic plasticity. *Neurobiol. Learn. Mem.* 89 599–603. 10.1016/j.nlm.2007.07.016 17881251 PMC2430891

[B112] Montalvo-Ortiz J. Cheng Z. Kranzler H. Zhang H. Gelernter J. (2019). Genomewide study of epigenetic biomarkers of opioid dependence in european- american women. *Sci. Rep.* 9: 4660. 10.1038/s41598-019-41110-7 30874594 PMC6420601

[B113] Moore L. Le T. Fan G. (2013). DNA methylation and its basic function. *Neuropsychopharmacology* 38 23–38. 10.1038/npp.2012.112 22781841 PMC3521964

[B114] Morozova T. Mackay T. Anholt R. (2014). Genetics and genomics of alcohol sensitivity. *Mol. Genet. Genomics* 289 253–269. 10.1007/s00438-013-0808-y 24395673 PMC4037586

[B115] Morris M. Monteggia L. (2014). Role of DNA methylation and the DNA methyltransferases in learning and memory. *Dialogues Clin. Neurosci.* 16 359–371. 10.31887/DCNS.2014.16.3/mmorris 25364286 PMC4214178

[B116] Moszczynska A. Burghardt K. J. Yu D. (2017). Neurotoxic doses of chronic methamphetamine trigger retrotransposition of the identifier element in rat dorsal dentate gyrus. *Genes* 8:96. 10.3390/genes8030096 28272323 PMC5368700

[B117] Nawarawong N. Olsen C. (2020). Within-animal comparisons of novelty and cocaine neuronal ensemble overlap in the nucleus accumbens and prefrontal cortex. *Behav. Brain Res.* 379 112275. 10.1016/j.bbr.2019.112275 31614186 PMC7937550

[B118] Nestler E. (2013). Cellular basis of memory for addiction. *Dialogues Clin. Neurosci.* 15 431–443. 10.31887/DCNS.2013.15.4/enestler 24459410 PMC3898681

[B119] Nestler E. (2014). Epigenetic mechanisms of drug addiction. *Neuropharmacology* 76 259–268. 10.1016/j.neuropharm.2013.04.004 23643695 PMC3766384

[B120] Nielsen D. Huang W. Hamon S. Maili L. Witkin B. Fox R. (2012a). Forced abstinence from cocaine self-administration is associated with dna methylation changes in myelin genes in the corpus callosum: A preliminary study. *Front. Psychiatry* 3:60. 10.3389/fpsyt.2012.00060 22712019 PMC3374938

[B121] Nielsen D. Utrankar A. Reyes J. Simons D. Kosten T. (2012b). Epigenetics of drug abuse: predisposition or response. *Pharmacogenomics* 13 1149–1160. 10.2217/pgs.12.94 22909205 PMC3463407

[B122] Nielsen D. Yuferov V. Hamon S. Jackson C. Ho A. Ott J. (2009). Increased OPRM1 DNA methylation in lymphocytes of methadone-maintained former heroin addicts. *Neuropsychopharmacology* 34 867–873. 10.1038/npp.2008.108 18650805 PMC2778040

[B123] Niwa M. Nitta A. Mizoguchi H. Ito Y. Noda Y. Nagai T. (2007). A novel molecule &quot;shati&quot; is involved in methamphetamine-induced hyperlocomotion, sensitization, and conditioned place preference. *J. Neurosci.* 27 7604–7615. 10.1523/JNEUROSCI.1575-07.2007 17626222 PMC6672622

[B124] Northcutt A. Hutchinson M. Wang X. Baratta M. Hiranita T. Cochran T. (2015). DAT isn’t all that: Cocaine reward and reinforcement require Toll-like receptor 4 signaling. *Mol. Psychiatry* 20 1525–1537. 10.1038/mp.2014.177 25644383 PMC4523496

[B125] Novikova S. He F. Bai J. Cutrufello N. Lidow M. Undieh A. (2008). Maternal cocaine administration in mice alters DNA methylation and gene expression in hippocampal neurons of neonatal and prepubertal offspring. *PLoS One* 3:e1919. 10.1371/journal.pone.0001919 18382688 PMC2271055

[B126] Numachi Y. Shen H. Yoshida S. Fujiyama K. Toda S. Matsuoka H. (2007). Methamphetamine alters expression of DNA methyltransferase 1 mRNA in rat brain. *Neurosci. Lett.* 414 213–217. 10.1016/j.neulet.2006.12.052 17254711

[B127] Oertel B. Doehring A. Roskam B. Kettner M. Hackmann N. Ferreirós N. (2012). Genetic-epigenetic interaction modulates μ-opioid receptor regulation. *Hum. Mol. Genet.* 21 4751–4760. 10.1093/hmg/dds314 22875838

[B128] Oni-Orisan O. Dansereau L. Marsit C. Smith L. Neal C. Della Grotta S. (2021). DNA methylation in children with prenatal methamphetamine exposure and environmental adversity. *Pediatr. Res.* 89 1152–1156. 10.1038/s41390-020-1058-4 32663835 PMC7855315

[B129] Planques A. Kerner P. Ferry L. Grunau C. Gazave E. Vervoort M. (2021). DNA methylation atlas and machinery in the developing and regenerating annelid Platynereis dumerilii. *BMC Biol.* 19:148. 10.1186/s12915-021-01074-5 34340707 PMC8330077

[B130] Ploense K. Li X. Baker-Andresen D. Carr A. Woodward N. Bagley J. (2018). Prolonged-access to cocaine induces distinct Homer2 DNA methylation, hydroxymethylation, and transcriptional profiles in the dorsomedial prefrontal cortex of Male Sprague-Dawley rats. *Neuropharmacology* 143 299–305. 10.1016/j.neuropharm.2018.09.029 30268522 PMC6371788

[B131] Radhakrishna U. Sadhasivam S. Radhakrishnan R. Forray A. Muvvala S. Metpally R. (2024). Placental cytochrome P450 methylomes in infants exposed to prenatal opioids: Exploring the effects of neonatal opioid withdrawal syndrome on health horizons. *Front. Genet.* 14:1292148. 10.3389/fgene.2023.1292148 38264209 PMC10805101

[B132] Radhakrishna U. Vishweswaraiah S. Uppala L. Szymanska M. Macknis J. Kumar S. (2021). Placental DNA methylation profiles in opioid-exposed pregnancies and associations with the neonatal opioid withdrawal syndrome. *Genomics* 113 1127–1135. 10.1016/j.ygeno.2021.03.006 33711455

[B133] Reilly M. Noronha A. Goldman D. Koob G. (2017). Genetic studies of alcohol dependence in the context of the addiction cycle. *Neuropharmacology* 122 3–21. 10.1016/j.neuropharm.2017.01.017 28118990 PMC6233301

[B134] Riccomagno M. Hurtado A. Wang H. Macopson J. Griner E. Betz A. (2012). The RacGAP β2-Chimaerin selectively mediates axonal pruning in the hippocampus. *Cell* 149 1594–1606. 10.1016/j.cell.2012.05.018 22726444 PMC3395473

[B135] Riyahi J. Taslimi Z. Gelfo F. Petrosini L. Haghparast A. (2024). Trans-generational effects of parental exposure to drugs of abuse on offspring memory functions. *Neurosci. Biobehav. Rev.* 160:105644. 10.1016/j.neubiorev.2024.105644 38548003

[B136] Robbins T. Ersche K. Everitt B. (2008). Drug addiction and the memory systems of the brain. *Ann. N. Y. Acad. Sci.* 1141 1–21. 10.1196/annals.1441.020 18991949

[B137] Robinson T. Kolb B. (1999). Alterations in the morphology of dendrites and dendritic spines in the nucleus accumbens and prefrontal cortex following repeated treatment with amphetamine or cocaine. *Eur. J. Neurosci.* 11 1598–1604. 10.1046/j.1460-9568.1999.00576.x 10215912

[B138] Rudenko A. Dawlaty M. Seo J. Cheng A. Meng J. Le T. (2013). Tet1 is critical for neuronal activity-regulated gene expression and memory extinction. *Neuron* 79 1109–1122. 10.1016/j.neuron.2013.08.003 24050401 PMC4543319

[B139] Sadri-Vakili G. (2015). Cocaine triggers epigenetic alterations in the corticostriatal circuit. *Brain Res.* 1628 50–59. 10.1016/j.brainres.2014.09.069 25301690 PMC4387100

[B140] Sagarkar S. Bhat N. Sapre M. Dudhabhate B. Kokare D. Subhedar N. (2022). TET1-induced DNA demethylation in dentate gyrus is important for reward conditioning and reinforcement. *Mol. Neurobiol.* 59 5426–5442. 10.1007/s12035-022-02917-0 35705787

[B141] Salehzadeh S. Mohammadian A. Salimi F. (2020). Effect of chronic methamphetamine injection on levels of BDNF mRNA and its CpG island methylation in prefrontal cortex of rats. *Asian J. Psychiatr.* 48:101884. 10.1016/j.ajp.2019.101884 31830601

[B142] Salinas R. Connolly D. Song H. (2020). Invited review: Epigenetics in neurodevelopment. *Neuropathol. Appl. Neurobiol.* 46 6–27. 10.1111/nan.12608 32056273 PMC7174139

[B143] Sandoval-Sierra J. Salgado García F. Brooks J. Derefinko K. Mozhui K. (2020). Effect of short-term prescription opioids on DNA methylation of the OPRM1 promoter. *Clin. Epigenetics* 12:76. 10.1186/s13148-020-00868-8 32493461 PMC7268244

[B144] Santos-Toscano R. Arevalo M. Garcia-Segura L. Grassi D. Lagunas N. (2023). Interaction of gonadal hormones, dopaminergic system, and epigenetic regulation in the generation of sex differences in substance use disorders: A systematic review. *Front. Neuroendocrinol.* 71:101085. 10.1016/j.yfrne.2023.101085 37543184

[B145] Sarraf S. Stancheva I. (2004). Methyl-CpG binding protein MBD1 couples histone H3 methylation at lysine 9 by SETDB1 to DNA replication and chromatin assembly. *Mol. Cell.* 15 595–605. 10.1016/j.molcel.2004.06.043 15327775

[B146] Saxonov S. Berg P. Brutlag D. L. (2006). A genome-wide analysis of CpG dinucleotides in the human genome distinguishes two distinct classes of promoters. *Proc. Natl. Acad. Sci. U S A.* 103 1412–1417. 10.1073/pnas.0510310103 16432200 PMC1345710

[B147] Schuster R. Kleimann A. Rehme M. Taschner L. Glahn A. Groh A. (2017). Elevated methylation and decreased serum concentrations of BDNF in patients in levomethadone compared to diamorphine maintenance treatment. *Eur. Arch. Psychiatry Clin. Neurosci.* 267 33–40. 10.1007/s00406-016-0668-7 26801497

[B148] Seisenberger S. Andrews S. Krueger F. Arand J. Walter J. Santos F. (2012). The dynamics of genome-wide DNA methylation reprogramming in mouse primordial germ cells. *Mol. Cell.* 48 849–862. 10.1016/j.molcel.2012.11.001 23219530 PMC3533687

[B149] Shu C. Sosnowski D. Tao R. Deep-Soboslay A. Kleinman J. Hyde T. (2021). Epigenome-wide study of brain DNA methylation following acute opioid intoxication. *Drug Alcohol. Depend.* 221:108658. 10.1016/j.drugalcdep.2021.108658 33667780 PMC8026744

[B150] Song C. Szulwach K. Dai Q. Fu Y. Mao S. Lin L. (2013). Genome-wide profiling of 5-formylcytosine reveals its roles in epigenetic priming. *Cell* 153 678–691. 10.1016/j.cell.2013.04.001 23602153 PMC3657391

[B151] Souza M. Sanvicente-Vieira B. Zaparte A. Baptista T. Nagai M. Mangone F. (2023). Cocaine use disorder effects on blood oxytocin levels and OXTR DNA methylation. *Neurosci. Lett.* 816:137506. 10.1016/j.neulet.2023.137506 37778686

[B152] Stevenson T. Prendergast B. (2013). Reversible DNA methylation regulates seasonal photoperiodic time measurement. *Proc. Natl. Acad. Sci. U S A.* 110 16651–16656. 10.1073/pnas.1310643110 24067648 PMC3799317

[B153] Streck E. Gonçalves C. Furlanetto C. Scaini G. Dal-Pizzol F. Quevedo J. (1999). Mitochondria and the central nervous system: Searching for a pathophysiological basis of psychiatric disorders. *Braz. J. Psychiatry* 36 156–167. 10.1590/1516-4446-2013-1224 24845118

[B154] Sultan F. Wang J. Tront J. Liebermann D. Sweatt J. (2012). Genetic deletion of Gadd45b, a regulator of active DNA demethylation, enhances long-term memory and synaptic plasticity. *J. Neurosci.* 32 17059–17066. 10.1523/JNEUROSCI.1747-12.2012 23197699 PMC3518911

[B155] Sun X. Bernstein M. Meng M. Rao S. Sørensen A. Yao L. (2020). Functionally distinct neuronal ensembles within the memory engram. *Cell* 181 410–423.e17. 10.1016/j.cell.2020.02.055 32187527 PMC7166195

[B156] Swinford-Jackson S. Fant B. Wimmer M. Chan D. Knouse M. Sarmiento M. (2022). Cocaine-induced changes in sperm Cdkn1a methylation are associated with cocaine resistance in male offspring. *J. Neurosci.* 42 2905–2916. 10.1523/JNEUROSCI.3172-20.2022 35232758 PMC8985859

[B157] Szulwach K. Li X. Li Y. Song C. Wu H. Dai Q. (2011). 5-hmC-mediated epigenetic dynamics during postnatal neurodevelopment and aging. *Nat. Neurosci.* 14 1607–1616. 10.1038/nn.2959 22037496 PMC3292193

[B158] Tahiliani M. Koh K. Shen Y. Pastor W. Bandukwala H. Brudno Y. (2009). Conversion of 5-methylcytosine to 5-hydroxymethylcytosine in mammalian DNA by MLL partner TET1. *Science* 324 930–935. 10.1126/science.1170116 19372391 PMC2715015

[B159] Takai D. Jones P. (2003). The CpG island searcher: A new WWW resource. *Silico Biol.* 3 235–240. 12954087

[B160] Tian W. Wang J. Zhang K. Teng H. Li C. Szyf M. (2016). Demethylation of c-MYB binding site mediates upregulation of Bdnf IV in cocaine-conditioned place preference. *Sci. Rep.* 6:22087. 10.1038/srep22087 26912258 PMC4766519

[B161] Tian W. Zhao M. Li M. Song T. Zhang M. Quan L. (2012). Reversal of cocaine-conditioned place preference through methyl supplementation in mice: Altering global DNA methylation in the prefrontal cortex. *PLoS One* 7:e33435. 10.1371/journal.pone.0033435 22438930 PMC3306398

[B162] Tomaselli D. Lucidi A. Rotili D. Mai A. (2020). Epigenetic polypharmacology: A new frontier for epi-drug discovery. *Med. Res. Rev.* 40 190–244. 10.1002/med.21600 31218726 PMC6917854

[B163] Townsel C. Truax B. Quaid M. Covault J. Dolinoy D. Goodrich J. (2024). Increased risk of severe neonatal opioid withdrawal syndrome in pregnancies with low placental ABCB1 DNA methylation. *J. Perinatol.* Online ahead of print. 10.1038/s41372-024-02060-9 39033231 PMC11743817

[B164] Trivedi M. Shah J. Hodgson N. Byun H. Deth R. (2014). Morphine induces redox-based changes in global DNA methylation and retrotransposon transcription by inhibition of excitatory amino acid transporter type 3-mediated cysteine uptake. *Mol. Pharmacol.* 85 747–757. 10.1124/mol.114.091728 24569088 PMC3990020

[B165] Turek-Plewa J. Jagodziński P. (2005). The role of mammalian DNA methyltransferases in the regulation of gene expression. *Cell. Mol. Biol. Lett.* 10 631–647. 16341272

[B166] Uno K. Miyazaki T. Sodeyama K. Miyamoto Y. Nitta A. (2017). Methamphetamine induces Shati/Nat8L expression in the mouse nucleus accumbens via CREB- and dopamine D1 receptor-dependent mechanism. *PLoS One* 12:e0174196. 10.1371/journal.pone.0174196 28319198 PMC5358781

[B167] Vassoler F. Byrnes E. Pierce R. (2014). The impact of exposure to addictive drugs on future generations: Physiological and behavioral effects. *Neuropharmacology* 76 269–275. 10.1016/j.neuropharm.2013.06.016 23810828 PMC3864776

[B168] Vassoler F. White S. Schmidt H. Sadri-Vakili G. Pierce R. (2013). Epigenetic inheritance of a cocaine-resistance phenotype. *Nat. Neurosci.* 16 42–47. 10.1038/nn.3280 23242310 PMC3531046

[B169] Veerasakul S. Watiktinkorn P. Thanoi S. Dalton C. Fachim H. Nudmamud-Thanoi S. (2017). Increased DNA methylation in the parvalbumin gene promoter is associated with methamphetamine dependence. *Pharmacogenomics* 18 1317–1322. 10.2217/pgs-2016-0188 28835159

[B170] Veremeyko T. Siddiqui S. Sotnikov I. Yung A. Ponomarev E. D. (2013). IL-4/IL-13-dependent and independent expression of miR-124 and its contribution to M2 phenotype of monocytic cells in normal conditions and during allergic inflammation. *PLoS One* 8:e81774. 10.1371/journal.pone.0081774 24358127 PMC3864800

[B171] Vidaki A. Daniel B. Court D. (2013). Forensic DNA methylation profiling–potential opportunities and challenges. *Forensic Sci. Int. Genet.* 7 499–507. 10.1016/j.fsigen.2013.05.004 23948320

[B172] Vissers C. Sinha A. Ming G. Song H. (2020). The epitranscriptome in stem cell biology and neural development. *Neurobiol. Dis.* 146:105139. 10.1016/j.nbd.2020.105139 33065280 PMC7686257

[B173] Wachman E. Hayes M. Lester B. Terrin N. Brown M. Nielsen D. (2014). Epigenetic variation in the mu-opioid receptor gene in infants with neonatal abstinence syndrome. *J. Pediatr.* 165 472–478. 10.1016/j.jpeds.2014.05.040 24996986 PMC4145036

[B174] Waddington C. (1942). The epigenotype. 1942. *Int. J. Epidemiol.* 41 10–13. 10.1093/ije/dyr184 22186258

[B175] Wang Y. Liu H. Sun Z. (2017). Lamarck rises from his grave: Parental environment-induced epigenetic inheritance in model organisms and humans. *Biol. Rev. Camb. Philos. Soc.* 92 2084–2111. 10.1111/brv.12322 28220606

[B176] Weinhold B. (2006). Epigenetics: The science of change. *Environ. Health Perspect.* 114 A160–A167. 10.1289/ehp.114-a160 16507447 PMC1392256

[B177] White S. Vassoler F. Schmidt H. Pierce R. Wimmer M. (2016). Enhanced anxiety in the male offspring of sires that self-administered cocaine. *Addict. Biol.* 21 802–810. 10.1111/adb.12258 25923597 PMC4626434

[B178] Wimmer M. Briand L. Fant B. Guercio L. Arreola A. Schmidt H. (2017). Paternal cocaine taking elicits epigenetic remodeling and memory deficits in male progeny. *Mol. Psychiatry* 22 1641–1650. 10.1038/mp.2017.8 28220045 PMC5568460

[B179] Wisor J. Schmidt M. Clegern W. (2011). Cerebral microglia mediate sleep/wake and neuroinflammatory effects of methamphetamine. *Brain Behav. Immun.* 25 767–776. 10.1016/j.bbi.2011.02.002 21333736

[B180] Wright K. Hollis F. Duclot F. Dossat A. Strong C. Francis T. (2015). Methyl supplementation attenuates cocaine-seeking behaviors and cocaine-induced c-Fos activation in a DNA methylation-dependent manner. *J. Neurosci.* 35 8948–8958. 10.1523/JNEUROSCI.5227-14.2015 26063926 PMC4461693

[B181] Yao H. Yang Y. Kim K. Bethel-Brown C. Gong N. Funa K. (2010). Molecular mechanisms involving sigma receptor-mediated induction of MCP-1: Implication for increased monocyte transmigration. *Blood* 115 4951–4962. 10.1182/blood-2010-01-266221 20354174 PMC2890169

[B182] Youngson N. Whitelaw E. (2008). Transgenerational epigenetic effects. *Annu. Rev. Genomics Hum. Genet.* 9 233–257. 10.1146/annurev.genom.9.081307.164445 18767965

[B183] Yuka K. Nishizawa D. Hasegawa J. Uno K. Miyanishi H. Ujike H. (2020). A single medical marker for diagnosis of methamphetamine addiction - DNA methylation of SHATI/NAT8L promoter sites from patient blood. *Curr. Pharm. Des.* 26 260–264. 10.2174/1381612826666200110111703 31924153

[B184] Zhang H. Lang Z. Zhu J. (2018). Dynamics and function of DNA methylation in plants. *Nat. Rev. Mol. Cell. Biol.* 19 489–506. 10.1038/s41580-018-0016-z 29784956

[B185] Zhang J. Han J. Sui N. (2014). Okadaic acid blocks the effects of 5-aza-2-deoxycytidine on consolidation, acquisition and retrieval of morphine-induced place preference in rats. *Neuropharmacology* 86 282–293. 10.1016/j.neuropharm.2014.08.005 25139850

[B186] Zhang J. Jiang F. Zheng W. Duan Y. Jin S. Shen F. (2020). DNMT3a in the hippocampal CA1 is crucial in the acquisition of morphine self-administration in rats. *Addict. Biol.* 25:e12730. 10.1111/adb.12730 30950138

[B187] Zhao Q. Hou J. Chen B. Shao X. Zhu R. Bu Q. (2015). Prenatal cocaine exposure impairs cognitive function of progeny via insulin growth factor II epigenetic regulation. *Neurobiol. Dis.* 82 54–65. 10.1016/j.nbd.2015.05.014 26054440

[B188] Zou Y. Sun X. Yang Q. Zheng M. Shimoni O. Ruan W. (2022). Blood-brain barrier-penetrating single CRISPR-Cas9 nanocapsules for effective and safe glioblastoma gene therapy. *Sci. Adv.* 8:eabm8011. 10.1126/sciadv.abm8011 35442747 PMC9020780

